# Autoantibodies elicited with SARS-CoV-2 infection are linked to alterations in double negative B cells

**DOI:** 10.3389/fimmu.2022.988125

**Published:** 2022-09-05

**Authors:** Moriah J. Castleman, Megan M. Stumpf, Nicholas R. Therrien, Mia J. Smith, Kelsey E. Lesteberg, Brent E. Palmer, James P. Maloney, William J. Janssen, Kara J. Mould, J. David Beckham, Roberta Pelanda, Raul M. Torres

**Affiliations:** ^1^ Department of Immunology and Microbiology, University of Colorado School of Medicine, Aurora, CO, United States; ^2^ Barbara Davis Center for Diabetes, Department of Pediatrics, University of Colorado School of Medicine, Aurora, CO, United States; ^3^ Department of Medicine, Division of Infectious Disease, University of Colorado School of Medicine, Aurora, CO, United States; ^4^ Department of Medicine, Division of Allergy and Clinical Immunology, University of Colorado School of Medicine, Aurora, CO, United States; ^5^ Department of Medicine, Division of Pulmonary Sciences and Critical Care Medicine, University of Colorado School of Medicine, Aurora, CO, United States; ^6^ Department of Medicine, National Jewish Health, Denver, CO, United States; ^7^ Department of Medicine, University of Colorado, Aurora, CO, United States; ^8^ Rocky Mountain Regional Veterans affairs (VA), Medical Center, Aurora, CO, United States

**Keywords:** double negative, DN1, DN2, DN3, B cells, inflammation, SARS–CoV–2, autoreactive

## Abstract

Double negative (DN) B cells (CD27-IgD-) comprise a heterogenous population of DN1, DN2, and the recently described DN3 and DN4 subsets. In autoimmune disease, DN2 cells are reported to be precursors to autoreactive antibody secreting cells and expansion of DN2 cells is linked to elevated interferon levels. Severe SARS-CoV-2 infection is characterized by elevated systemic levels of pro-inflammatory cytokines and serum autoantibodies and expansion of the DN2 subset in severe SARS-CoV-2 infection has been reported. However, the activation status, functional capacity and contribution to virally-induced autoantibody production by DN subsets is not established. Here, we validate the finding that severe SARS-CoV-2 infection is associated with a reduction in the frequency of DN1 cells coinciding with an increase in the frequency of DN2 and DN3 cells. We further demonstrate that with severe viral infection DN subsets are at a heightened level of activation, display changes in immunoglobulin class isotype frequency and have functional BCR signaling. Increases in overall systemic inflammation (CRP), as well as specific pro-inflammatory cytokines (TNFα, IL-6, IFNγ, IL-1β), significantly correlate with the skewing of DN1, DN2 and DN3 subsets during severe SARS-CoV-2 infection. Importantly, the reduction in DN1 cell frequency and expansion of the DN3 population during severe infection significantly correlates with increased levels of serum autoantibodies. Thus, systemic inflammation during SARS-CoV-2 infection drives changes in Double Negative subset frequency, likely impacting their contribution to generation of autoreactive antibodies.

## Graphical Abstract

Created with BioRender.com.

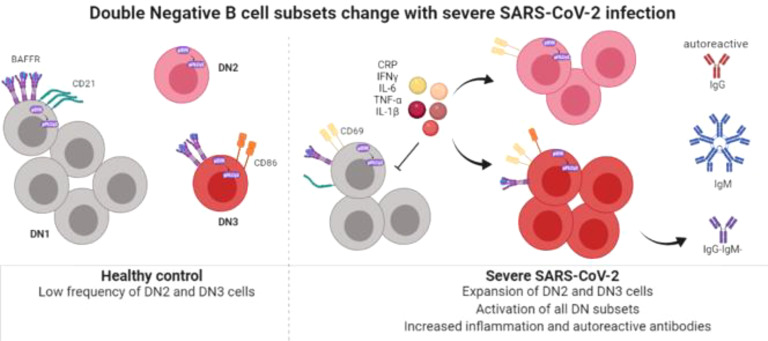

## Introduction

B lymphocytes from human peripheral blood can be categorized (based on the expression of IgD and CD27 surface receptors) into naive (CD27-IgD+), unswitched memory (CD27+IgD+) and Ig class-switched memory (CD27+ IgD-), or Double-Negative (DN: CD27-IgD-) B cell subsets (1, 2). DN B cells were first identified due to their expansion in patients with Systemic Lupus Erythematosus (SLE) and are considered memory B cells due to the similarity in phenotype with conventional memory B cells, presence of class-switched immunoglobulins IgG or IgA, and evidence of somatic hypermutation indicating DN cells are antigen experienced (3–5). In addition to SLE, expansion of the Double Negative population has been reported in a variety of autoimmune disorders including; Guillain-Barre syndrome, Myasthenia gravis and Multiple sclerosis (6, 7), as well as, Common Variable Immunodeficiency (CVID) where an expansion in the autoreactive VH4-34 DN population was also reported (8). Furthermore, expansion of DN B cells in SLE patients correlated with higher titers of serum VH4-34 autoreactive antibodies (4, 5). Together these reports suggest a contribution of DN cells to autoimmunity.

Further examination of SLE patients revealed that Double Negative B cells are a heterogenous population of cells comprised of DN1 and DN2 subsets identified based not only on CD27^-^IgD^-^ but also on differential expression of CD11c and CD21, whereby DN1 cells express CD21 but not CD11c (CD21^+^CD11c^–^) and DN2 cells express high levels of CD11c in the absence of CD21 (CD21^–^CD11c^++^) (2, 9). In SLE flares, there is a loss of DN1 cells with a corresponding increase in DN2 cells, with DN2 cells described as a pathogenic precursor to autoreactive antibody secreting cells (9). Single cell transcriptomic analysis of PBMCs from healthy controls has suggested the existence of two additional DN subsets called DN3 and DN4 cells, whereby DN3 cells were enriched in *IGHA2* transcripts and DN4 cells were enriched in *IGHE* transcripts (10). More recently, cellular evidence confirming the existence of a DN3 subset lacking expression of both CD11c and CD21 has been reported (CD11c^-^CD21^-^), but there is limited evidence for the existence of a DN4 subset expressing both CD11c and CD21 (11–13). The functional role of these diverse Double Negative subsets in various immune responses, particularly in the context of viral infection, and the mechanisms that promote generation of each unique subset compared to another remain to be determined.

Given their relatively recent identification, there is limited information on the B cell developmental pathways that populate the DN3 and DN4 subsets. However, for the DN2 subset a role for inflammatory cytokines in modulating their development has been established. Specifically, increased frequencies of DN2 cells in SLE patients were correlated with increased levels of IFN-γ, IFN-λ, and IFN-γ-induced cytokines including TNF-α and IL-6 (9, 14, 15). Accordingly, *in vitro* generation of DN2 cells from naive B cell precursors can be facilitated by either IFN-γ or IFN-λ in the presence of TLR7L, IL-21, BAFF and BCR stimulation, a process that could be inhibited by IL-4 and CD40L mimicking T cell help (9, 14, 15). Together, these reports suggest a role for inflammatory cytokines, such as is typically induced during viral infection, in regulating the composition of the Double Negative population.

Severe acute respiratory syndrome coronavirus 2 (SARS-CoV-2), the causative agent of the current coronavirus disease 2019 (COVID-19) has a multi-faceted immunopathology including T cell activation, increased IFN-γ, TNF-α, IL-6, IL-1β cytokines and production of autoreactive antibodies (16–20). Additionally, multiple groups have reported an expansion of the DN2 and DN3 subsets in PBMCs from severe SARS-CoV-2 infection (11, 12, 21, 22) and a concordant reduction in the DN1 subset (11, 12). Stratification of severely infected samples into those with high and low levels of C-reactive protein (CRP), an indicator of overall systemic inflammation, revealed that expansion of DN2 and DN3 cells was more predominant in high CRP samples, implicating inflammation as a driver of DN2/3 subset expansion with viral infection (21). Expansion of the DN3 subset correlated with a variety of clinical parameters including increased respiratory rate and increased levels of D-dimer and CRP (11), suggesting a possible role for DN3 cells in disease pathogenesis. Furthermore, individuals who recovered from infection had a higher frequency of DN1 cells than individuals who succumbed to infection (23), indicating DN1 cells may play a protective role in viral infection. Despite these reports on DN subset frequency and correlation with disease parameters, there is still limited information on DN cells in both healthy controls and during SARS-CoV-2 infection. Specifically, the phenotype of DN subsets with regard to activation and inhibitory receptor expression as well as responses to BCR signaling (to determine functionality of the BCR) have not been reported for DN subsets in SARS-CoV-2 infection nor has the association of DN subsets with autoreactive antibody production in viral infection been reported. Therefore, the phenotype, function and possible contribution to virally-induced autoimmunity by DN subsets, particularly the novel DN3 population, needs further characterization.

In this report, we have interrogated PBMCs and plasma from healthy controls, individuals immunized against SARS-CoV-2 by mRNA vaccines, and individuals with mild or severe SARS-CoV-2 infection. The results of these analyses confirm that there is a reduction in the frequency of DN1 cells within the Double Negative population coinciding with an increase in the frequency of DN2 and DN3 cells in severe SARS-CoV-2 patients. With severe viral infection, the B cells within each DN subset are at a heightened level of activation, display changes in immunoglobulin class isotype frequency and possess the ability to signal through the BCR. Importantly, increases in overall systemic inflammation (CRP), as well as increases in specific pro-inflammatory cytokines (TNFα, IL-6, IFNγ, IL-1β), significantly correlate with the alteration in the frequencies of DN1, DN2 and DN3 subsets during severe SARS-CoV-2 infection. Furthermore, we show that the reduction in DN1 cells and expansion of DN3 cells is significantly correlated with increases in relative titer of autoreactive antibodies during severe infection. Together these data provide evidence that systemic inflammation during SARS-CoV-2 infection likely drives changes in Double Negative subset frequency, thereby impacting their contribution to generation of autoreactive antibodies.

## Materials and methods

### Human peripheral blood mononuclear cells

Individuals with severe SARS-CoV-2 infection were hospitalized at the University of Colorado Hospital (UCH) or St. Joseph’s Hospital (SJH). Informed consent to donate whole blood was obtained from a legally authorized representative (SJH) or by an approved waiver of consent (UCH). Subjects were 18 years of age or older and mechanically ventilated for acute respiratory distress syndrome, as defined by the Berlin Criteria, due to SARS-CoV-2. The presence of virus was confirmed by polymerase chain reaction of a nasal swab. Patients were excluded from this study if they had a history of solid organ or bone marrow transplants, chronic lung disease, hemoptysis, increased risk for bleeding, pregnancy or who were immunosuppressed. We did not actively exclude patients with a diagnose of autoimmune disease prior to acquisition of severe SARS-CoV-2 infection, however upon retrospective review of patient files, 13 out of 14 patients did not have an autoimmune disease and 1 out of 14 patients had Type 1 Diabetes Mellitus. Whole blood was collected from central venous catheters in cell preparation tubes with sodium citrate and processed per the manufacturer’s instructions (BD Biosciences, San Jose, CA). Plasma was stored at -80 degrees until use. PBMCs were resuspended in 90% FBS with 10% DMSO or 90% FBS + DMEM and 10% DMSO and stored in liquid nitrogen until use.

For individuals immunized against SARS-CoV-2 by Pfizer BNT162b2-mRNA or Moderna mRNA-1273, informed consent was provided for the acquisition of whole blood as well as the dates of their primary inoculation and booster. For individuals deemed mildly infected (convalescent stage) with SARS-CoV-2, informed consent was provided for the acquisition of whole blood, and they were included in this study if they had a positive viral qPCR test or the presence of anti-SARS-CoV2 antibodies in the absence of vaccination (samples were collected before vaccination was available to the general public) and did not require hospitalization. For these individuals, whole blood was drawn by staff at the University of Colorado Clinical and Translation Research Centers (CTRC), part of the Colorado Clinical and Translation Sciences Institute (CCTSI), in sodium heparin tubes (BD, Franklin Lakes, NJ). Whole blood was centrifuged at 1,700 rpm for 5minutes to collect plasma. Plasma was stored at -80°C until use. Cells were then diluted in 1X PBS, suspended over a Ficoll-Paque gradient and centrifuged 2,400 rpm for 25 minutes to isolate PBMCs from the buffy coat. PBMCs were washed in 1X PBS, and enumerated by hemocytometer. PBMCs were resuspended in 90% FBS with 10% DMSO and stored in liquid nitrogen until use.

For healthy control samples, plateletpheresis leukoreduction filter (LRS chambers) were purchased from Vitalant Blood Center (Denver, CO). Cells were diluted in media containing RPMI Medium 1640 (Gibco, Netherlands) with 10% FBS, 1mM sodium pyruvate (Gibco), 1X non-essential amino acids (Gibco), 1X Glutamax (Gibco) and 50uM 2-mercaptoethanol (Sigma-Aldrich, St. Louis, Missouri). Cells were then suspended over a Ficoll- Paque gradient (Cytiva, Sweden) and centrifuged 2,400 rpm for 25 minutes at RT to isolate PBMCs in the buffy coat layer. PBMCs were washed in media, the cell pellet was resuspended in 1X PBS (Corning, Glendale, Arizona) and cell count was enumerated by hemocytometer. PBMCs were resuspended in 90% FBS (Gemini, Sacramento, CA) with 10% DMSO (ATCC, Manassas, VA) and stored in liquid nitrogen until use. LRS chambers do not allow the acquisition of plasma, thus when plasma was examined in this study, the immunized individuals were used as a comparator to mild or severe SARS-CoV-2 individuals.

The Colorado Multiple Institutional Review Board (COMIRB) at the University of Colorado School of Medicine and National Jewish Health approved the use of human plasma and PBMCs, and this study was performed under the Declaration of Helsinki.

### Frequency and phenotyping of B cell subsets *ex vivo* by flow cytometry

PBMCs were quickly thawed in a 37°C water bath, washed in warm media containing RPMI Medium 1640 with 25mM HEPES (Corning), centrifuged 1,500 rpm x 5min at RT, washed again and enumerated by hemocytometer. PBMCs were stained for 20min on ice in 1X PBS with the following antibodies (clone in parenthesis): CD3 (OKT3), CD19 (SJ25C1), CD27 (M-T271), IgD (IA6-2), CD21 (Bu32), CD11c (B-ly6), FcRL5 (509f6), IgM (MHM-88), CD69 (FN50), CD86 (IT2.2), CD72 (REA231), CD22 (HIB22), BAFFR (11C1) and IgG (G18-145) and in the presence of Live/Dead Blue for UV viability dye (Invitrogen, Eugene, OR). Cells were then washed in 1X PBS, centrifuged 1,500 rpm x 5 min at 4°C, fixed in 4% PFA (Fisher, Fair Lawn, NJ) for 15min at RT, washed with 1X PBS and resuspended in FACS Buffer containing 1% BSA (Fisher, Fair Lawn, NJ) + 0.05% Sodium Azide (Aldrich, St. Louis, MO) in 1X PBS.

All flow cytometry data was acquired on the Cytek Aurora. PBMCs from healthy humans were used for single color reference controls except where the use of Ultra Comp eBeads (Invitrogen) was necessary. Flow cytometry data was analyzed using FlowJo software (v 10.8.1). Within the lymphocyte single cell viable gates, DN1 cells were identified as CD3- CD19+ CD27- IgD- CD21+ CD11c-, DN2 cells were identified as CD3- CD19+ CD27- IgD- CD21- CD11c++, and DN3 cells were identified as CD3- CD19+ CD27- IgD- CD21- CD11c- in accordance with other groups (2, 11, 12). The frequency of DN1, DN2 or DN3 subsets were enumerated out of the total Double negative population (CD27-IgD-) or out of the total B cell population (CD19+). Expression of surface markers on each DN subset are reported as geometric mean fluorescent intensity (gMFI). FMOs used as staining controls are indicated in figure legends.

### Assessment of BCR signaling by phospho flow cytometry

PBMCs were thawed as described above. After enumeration, PBMCs were incubated for 45min at 37°C+ 5% CO2 to reduce basal phosphorylation levels in warm serum-free RMPI Medium 1640 while in the prescence of the following antibodies (clone): CD3 (OKT3), CD19 (SJ25C1), CD27 (M-T271), CD21 (Bu32), CD11c (B-ly6), and Live/Dead Blue for UV viability dye. Cells were centrifuged 1,500 rpm x 5min at RT, resuspended in warm RPMI with 5% FBS and stimulated with either 10ug/mL Rabbit anti-human IgG (H+L) F(ab’)2 (Southern Biotech, Birmingham, AL) or 75μM pervanadate (used as an experimental positive control) for 5 minutes in a 37°C water bath. After a quick centrifuge spin to pellet the cells (2,400 g x 30 seconds at RT), PBMCs were resuspended in 100uL per million cells Cytofix/Cytoperm (BD) and incubated for 20 minutes on ice. Cells were washed in 1X Perm/Wash (BD), then incubated in the presence of this buffer for intracellular staining for 30min on ice with the following antibodies (clone): pSYK Y348 (I120-722), pPLCγ2 Y759 (K86-689.37) and IgD (IA6-2). Cells were washed with 1X Perm/Wash, centrifuged 1,500 rpm x 5min at 4°C, and resuspended in FACS Buffer containing 1% BSA (Fisher)+ 0.05% Sodium Azide (Aldrich) in 1X PBS. Data was acquired on the Cytek Aurora and cell subsets defined as described above.

### Quantification of cytokines in plasma

Plasma was thawed on ice and TNFα, IL-6, IFNγ, and IL-1β were quantified by the U-PLEX Assay and CRP was quantified using the V-PLEX Assay according to manufacturer’s instructions (Meso Scale Discovery, Rockville, MD). Both assays were evaluated on the QuickPlex SQ 120 Instrument (Meso Scale Discovery).

### Measurement of autoreactive IgG antibodies in plasma

Autoreactive IgG antibodies were enumerated in plasma samples as previously described (24). Briefly, 96-well Nunc-Immuno MaxiSorp plates (Thermo Fisher Scientific) were either coated for 2 hours at 37°C with 0.5ug/mL 9G4 antibody to identify VH4-34 (kind gift of Dr. John Cambier, University of Colorado School of Medicine), coated overnight at 4°C with either 15ug/mL of sonicated calf thymus DNA for chromatin (Sigma-Aldrich), 1ug/mL Smith antigen (Arotec Diagnostics), or 50ug/mL cardiolipin (Sigma-Aldrich). The 9G4 monoclonal antibody is specific to the VH4-34 idiotype and can be used to identify B cells expressing the VH4-34 BCR (25, 26). Plates were washed 3X, blocked for 1-2 hour at 37°C. To block plates for detection of anti-chromatin, anti-Smith or VH4-34 autoantibodies, a buffer of 1X PBS, 1mM EDTA, 0.05% NaN3 and 1% BSA was used. To block plates for detection of anti-cardiolipin autoantibodies a blocking buffer of 1X PBS with 1% BSA was used. For 9G4 reactivity, each plasma sample was plated starting at 1:200 in a 3-fold serial dilution across 6 dilutions and incubated for overnight at 4°C. For chromatin, Smith or cardiolipin reactivity, each plasma sample was plated starting at 1:8 in a 3-fold serial dilution across 6 dilutions and incubated for 2 hours at 37°C. Plates were washed 3X and incubated with Goat anti-human IgG (for 9G4 Goat anti-human IgG multispecies cross absorbed was used) conjugated to alkaline phosphatase (Southern Biotech) for 1 hour at 37°C. Plates were washed 3X and developed with 1 mg/ml of 4-nitrophenyl phosphate disodium salt hexahydrate (Alkaline Phosphatase Substrate; Sigma-Aldrich) diluted in developing buffer (1M diethanolamine, 8.4 mM MgCl2, and 0.02% NaN3 in water) and incubated at 37°C for 10-30 minutes. Absorbance values (O.D.) were read at 405 nm on the VersaMax ELISA reader (MDS Analytical Technologies). The dilutions were log transformed to generate a curve and the linear part of the curve was used to select a dilution at which to compare relative O.D. values for each group. For absolute titers of chromatin, Smith or cardiolipin reactivity O.D. values were compared at 1/128 dilution and for 9G4 reactivity O.D. values were compared at 1/16200 dilution. The absolute titers were then normalized by the total amount of IgG in the corresponding plasma sample (quantification of total IgG described below).

### Quantification of total IgG in plasma

Levels of total IgG in plasma were quantified as previously described (24). Briefly, 96-well plates were coated with Goat anti-human IgG capture antibody (Southern Biotech) overnight at 4°C in 1X PBS. Plates were then washed 3X and blocked for 1 hour at 37°C. Plasma samples were plated starting at a 1:200 dilution in a 5-fold serial dilution across 6 points and incubated for 2 hours at 37°C. A standard curve of IgG (Southern Biotech) starting at 1pg/mL was used to quantify total levels of IgG in each plasma sample. Plates were washed 3X and incubated with Goat anti-human IgG conjugated to alkaline phosphatase (Southern Biotech) in 1X PBS for 1 hour at 37°C. Plates were washed and developed as described above. For absolute titers of IgG O.D. values were compared at 1/12500.

### Data analysis

Data was graphed and analyzed using Prism Graph-Pad Software (v 9.2.0). One-way ANOVA was used to determine the significance of differences between cohorts (healthy controls, immunized samples, mild or severe infection) or between cell subsets (DN1, DN2 and DN3) as indicated in the figure legends. A Pearson correlation was used to determine the significance of differences between systemic cytokines with the frequency of DN subsets or between the frequency of DN subsets with the titer of autoreactive antibodies as indicated in figure legends. Significance was defined as p< 0.05. If a trend to significance p<0.09 was observed, then it was noted on the figure. Each human donor in a group is represented by a dot on the scatter plot and the total sample size measured for each assay is indicated in each figure legend.

## Results

### Altered frequencies of Double Negative B cell subsets during severe SARS-CoV-2 infection

**Figure 1:**
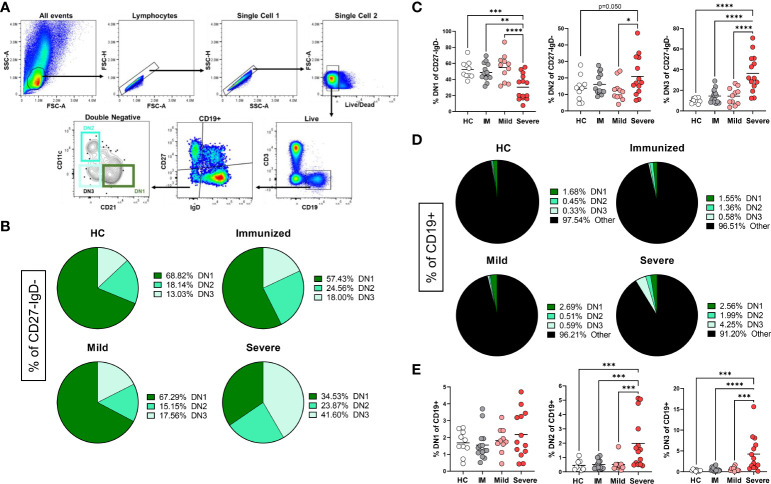
Alteration in the frequency of Double Negative subsets with Severe SARS-CoV-2 infection. **(A)** Flow plots demonstrating the gating strategy to identify DN1, DN2 and DN3 populations in human PBMCs. **(B)** Pie charts depicting the average frequency of DN1, DN2, and DN3 subsets within the total double negative population (CD27-IgD-) for healthy controls (HC), individuals immunized against SARS-CoV-2 (IM), individuals with mild or severe SARS-CoV-2 infection. **(C)** Quantification comparing the frequency of each DN subset within total double negative population (CD27-IgD-). **(D)** Pie charts depicting the average frequency of DN1, DN2, and DN3 subsets within the total B cell population (CD19+). **(E)** Quantification comparing the frequency of each DN subset within total B cells (CD19+) between healthy controls (HC, N=10), individuals immunized (IM, N=15) against SARS-CoV-2, and individuals with mild (N=11) or severe SARS-CoV-2 infection (N=14). Statistics: one-way ANOVA, *p < 0.05, **p < 0.01, ***p < 0.001, ****p < 0.0001.

To begin to characterize how immunization and viral infection might influence the presence of Double Negative B cell subsets, we collected human peripheral blood mononuclear cells (PBMCs) from healthy controls, individuals immunized with an mRNA vaccine against SARS-CoV-2, or individuals convalescing from a mild SARS-CoV-2 infection or subjects with severe SARS-CoV-2 infection requiring hospitalization (**Supplemental Table 1**). Double Negative (CD27^–^IgD^–^) B cells comprise a heterogeneous population of DN1, DN2 and DN3 subsets which are identified based on differential expression of CD11c and CD21 (**Figure 1A**), whereby DN1 cells express CD21 but not CD11c (CD21^+^CD11c^–^), DN2 cells expressing high levels of CD11c but not CD21 (CD21^–^CD11c^++^), and DN3 cells do not express either surface receptor (CD21^–^CD11c^–^) (2, 11, 12). A DN4 population expressing both CD11c and CD21, has been suggested based on transcriptome and phenotypic analyses (10, 11), however, in our analyses we did not observe a reproducibly distinct DN4 subset (CD21^+^CD11c^+^) in all individuals, thus this population was not further characterized. As expected (9), in healthy controls within the Double Negative population, DN1 cells were the most frequent subset followed by DN2 cells with DN3 cells being the most infrequent subset (**Figure 1B**). A similar hierarchy of DN subsets (DN1>DN2>DN3) within the DN population was also observed in individuals immunized against SARS-CoV-2 (**Figure 1B**). Conversely, individuals with mild SARS-CoV-2 infection harbored similar frequencies of DN3 and DN2 cells with DN1 cells remaining as the predominant subset (**Figure 1B**). Importantly, for individuals with severe SARS-CoV-2 infection, DN3 cells were the most frequent subset compared to DN1 and DN2 cells within the Double Negative population (**Figure 1B**).

Upon comparison of the frequency of subsets within the Double Negative population between healthy controls, individuals immunized against SARS-CoV-2, and subjects with either mild or severe SARS-CoV-2 infection demonstrated that there is a significant loss of DN1 cells with severe infection (**Figure 1C**), in keeping with previous reports (11, 12). A significant increase in DN2 cells within the Double Negative population with severe SARS-CoV-2 infection was observed as compared to DN2 cells from mild infection (**Figure 1C**). Similarly, in severe infection a significant increase in DN3 cells within the Double Negative population with severe infection was observed as compared to DN3 cells from healthy controls, individuals immunized against SARS-CoV-2 and subjects with mild SARS-CoV-2 infection (**Figure 1C**).

DN cells are a relatively minor population within the total B cell population (**Figure 1D**) and quantification of the DN1 subset did not reveal any significant differences in the frequency of DN1 cells amongst CD19+ cells with severe SARS-CoV-2 infection compared to other individuals (**Figure 1E**). Importantly, we observed a significant increase in DN2 and DN3 cells within the total B cell population with severe SARS-CoV-2 infection compared to heathy controls, individuals immunized against SARS-CoV-2 or subjects with mild infection (**Figure 1E**), in accordance with previous reports (22, 23).

Overall, these data demonstrate that the Double Negative population undergoes major alterations in subset frequency with severe SARS-CoV-2 infection suggesting that viral infection regulates the composition of DN1, DN2 and DN3 B cell subsets.

### Comparison of DN1, DN2, and DN3 cells in healthy individuals

**Figure 2:**
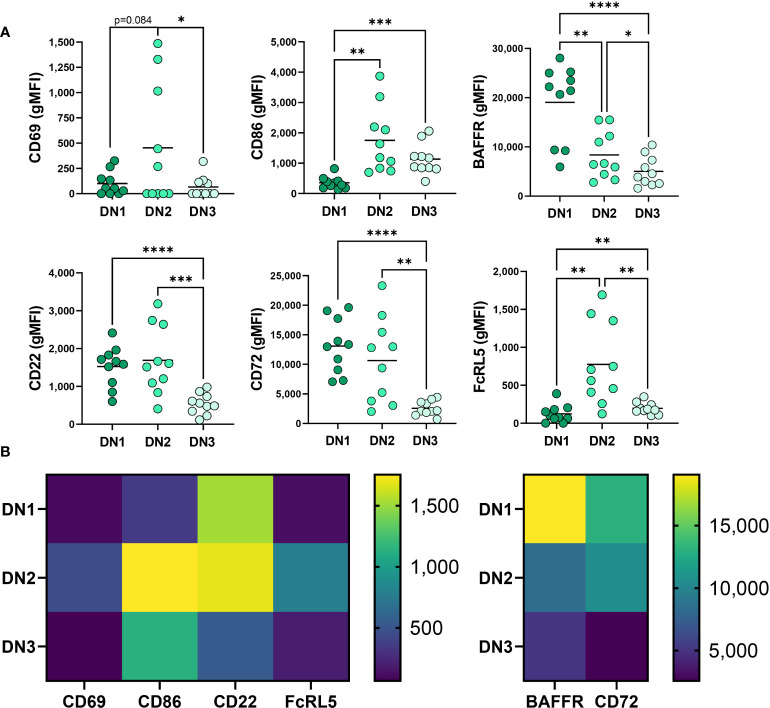
Phenotypic comparison of DN1, DN2 and DN3 cells in healthy controls. **(A)** Quantification of expression level (gMFI: geometric mean fluorescent intensity) of CD69, CD86, BAFFR, CD22, CD72 and FcRL5 on DN1, DN2 or DN3 cells from healthy controls (N=10) and **(B)** a heatmap visualizing the differences between DN1, DN2 and DN3 subsets of average gMFI values. Statistics: one-way ANOVA, *p < 0.05, **p < 0.01, ***p < 0.001, ****p < 0.0001.

To better characterize the uniqueness of B cell subsets within the Double Negative B cell population, especially the novel DN3 subset, we phenotypically compared the DN1, DN2 and DN3 subsets with each other from healthy controls. It was previously reported that DN2 cells from SLE patients express higher levels of the activation marker CD69 when compared to conventional memory B cells (9). In accord with that finding, our study revealed DN2 cells from healthy individuals have on average the highest level of CD69 expression compared to DN1 and DN3 cells in healthy controls (**Figure 2**), indicating DN2 cells naturally reside at a more activated state than other Double Negative subsets. In healthy controls, DN2 cells also have the highest expression level of CD86, a co-receptor that stimulates T cells (27), compared to DN1 or DN3 cells, with DN3 cells displaying a higher level of CD86 expression than DN1 cells (**Figure 2**). These data indicate that under normal conditions DN2 cells are poised to participate in B-T cell interactions, which was unexpected given DN2 cells are reported to participate in extrafollicular antibody responses (9), a process that includes T-cell independent antibody responses (28). Finally, our results further suggest DN3 cells are more likely to participate in activation of T cells through higher CD86 expression than DN1 cells.

Comparison of BAFFR expression, a receptor important for response to the survival cytokine BAFF (29), demonstrated that in healthy controls DN1 cells have significantly higher expression of BAFFR than DN2 and DN3 cells and DN2 cells express an intermediate level compared to the lowest BAFFR levels on DN3 cells (**Figure 2**). These data suggest that DN1 cells are the subset most dependent on BAFF for survival, whereas DN3 cells are the least dependent on BAFF for survival.

We also queried expression of receptors known to inhibit BCR signaling (30–32), specifically measuring CD72, CD22 and FcRL5 inhibitory receptor expression on Double Negative B cell subsets in healthy controls. DN2 cells from SLE patients were reported to express the highest level of CD22 when compared to conventional memory cells (9). Examination of CD22 demonstrated comparable CD22 expression between DN2 and DN1 cells in healthy controls (**Figure 2**) whereas DN3 cells expressed the lowest level of CD22 (**Figure 2**), implying that DN3 cells might experience the least CD22-mediated inhibition of BCR signaling *via* the recruitment of SHP-1 (33). Examination of another inhibitory receptor, CD72, revealed that DN1 cells have the highest level of CD72 expression compared to DN2 and DN3 cells, and DN3 cells have the lowest expression level of CD72 (**Figure 2**). These data suggest that in healthy controls CD72 may play an important role in regulating DN1 cells compared to other Double Negative B cell subsets. Finally, we compared expression of FcRL5 on Double Negative B cell subsets and demonstrate that in healthy controls DN2 cells express the highest level of FcRL5 (**Figure 2**), suggesting FcRL5 uniquely regulates the DN2 subset.

Together these findings begin to characterize the novel DN3 subset phenotype relative to the better characterized DN1 and DN2 subsets in healthy individuals with key features of DN3 cells expressing the lowest levels of CD22, CD72, CD69 and BAFFR out of the DN subsets.

### Phenotypic alteration in DN1 cells during severe SARS-CoV-2 infection

**Figure 3:**
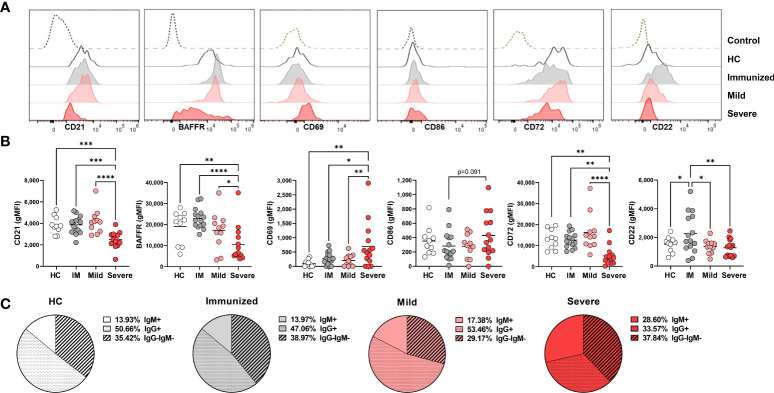
Phenotypic changes in DN1 cells with Severe SARS-CoV-2 infection. **(A)** Representative histograms depicting expression of CD21, BAFFR CD69, CD86, CD72, and CD22 on DN1 cells from a healthy control (HC), an individual immunized against SARS-CoV-2 or with mild or severe SARS-CoV-2 infection. Control dotted histogram is an FMO for CD69, CD68, CD72 and CD22. Control dotted histogram for BAFFR and CD21 is expression on CD3+ T cells from a healthy control subject. **(B)** Quantification of CD21, BAFFR, CD69, CD86, CD72, and CD22 expression (gMFI: geometric mean fluorescent intensity) on DN1 cells from healthy controls (HC), individuals immunized against SARS-CoV-2 (IM), or with mild or severe SARS-CoV-2 infection. **(C)** Pie charts depicting the average frequency of IgM+, IgG+, or IgG-IgM- DN1 cells for healthy controls (HC, N=10), individuals immunized against SARS-CoV-2 (N=15), or with mild (N=11) or severe SARS-CoV-2 infection (N=14). Statistics: one-way ANOVA, *p < 0.05, **p < 0.01, ***p < 0.001, ****p < 0.0001.

We next questioned how SARS-CoV-2 infection might promote a reduced frequency of DN1 cells and initially measured expression of CD21, the defining receptor on DN1 cells. This analysis revealed a significant reduction in CD21 expression on DN1 with severe SARS-CoV-2 infection as compared to DN1 cells from healthy controls, immunized individuals and those with mild SARS-CoV-2 infection (**Figures 3A, B**). These data suggest that with severe viral infection, loss of CD21 surface expression may account, at least in part, for the reduction in DN1 cell frequency. Examination of BAFFR on DN1 cells revealed that DN1 cells from severe SARS-CoV-2 infected subjects express significantly lower levels of BAFFR when compared to DN1 cells from healthy controls or individuals immunized against SARS-CoV-2 or with mild SARS-CoV-2 infection (**Figures 3A, B**). This would suggest DN1 cells inefficiently compete for BAFF and a subsequent reduction in DN1 cell survival may accompany severe viral infection and further account for the diminution of the DN1 population.

To assess the influence of viral infection on DN1 B cell activation, we evaluated expression of the CD69 and CD86 activation receptors on virally infected and control individuals. These analyses revealed that there was a significant increase in expression of CD69 on DN1 cells with severe SARS-CoV-2 infection as compared to healthy controls, immunized individuals or those who had experienced mild SARS-CoV-2 infection. We also observed a trend of higher CD86 expression on DN1 cells from severe SARS-CoV-2 infection as compared to DN1 cells from individuals immunized against SARS-CoV-2 (**Figures 3A, B**). Together these results indicate that DN1 cells are in an activated state with viral infection.

We also measured the levels of the CD22 and CD72 inhibitory receptors on DN1 B cells as an assessment of functional capacity since both inhibitory receptors are able to negatively regulate BCR signaling (30, 31). DN1 cells from severe SARS-CoV-2 infected subjects express significantly lower levels of CD72 compared to DN1 cells from healthy controls or individuals immunized against SARS-CoV-2 or with mild SARS-CoV-2 infection (**Figures 3A, B**). Interestingly, immunization against SARS-CoV-2 appeared to increase expression of CD22 on DN1 cells such that DN1 cells from healthy controls and individuals with mild or severe SARS-CoV-2 infection had significantly lower levels of CD22 expression than DN1 cells from immunized subjects (**Figures 3A, B**). These data indicate that immunization and viral infection differentially effect inhibitory receptor expression on DN1 cells and DN1 cells in severe infection express relatively reduced levels of both the CD72 and CD22 inhibitory receptors.

Since the more broadly-defined Double Negative B population was initially described as an antigen-experienced memory B cell population lacking expression of CD27 with nearly half of the population class-switched to IgG (5), and the majority of DN1 cells from heathy controls express IgG (9), we asked whether viral infection also modified isotype class dominance in DN1, DN2 and DN3 subsets. In healthy controls, the majority of DN1 cells are IgG^+^, a third of DN1 cells are IgG^–^IgM^–^ and the remaining small portion are IgM^+^ (**Figure 3C**). A similar breakdown of immunoglobulin isotype class was observed with DN1 cells from immunized individuals and subjects with mild SARS-CoV-2 infection (**Figure 3C**). Conversely, with DN1 cells from subjects with severe SARS-CoV-2 infection we observed a significant reduction in IgG^+^ cells and a corresponding increase in IgM^+^ DN1 cells (**Figure 3C**), suggesting that severe viral infection modifies immunoglobulin class type in DN1 cells.

Overall, these data demonstrate that DN1 are phenotypically altered with severe SARS-CoV-2 infection such that they are at a heightened level of activation and express reduced levels of inhibitory receptors and BAFFR and display a shift in antibody isotype class.

### Activation of DN2 cells during severe SARS-CoV-2 infection

**Figure 4:**
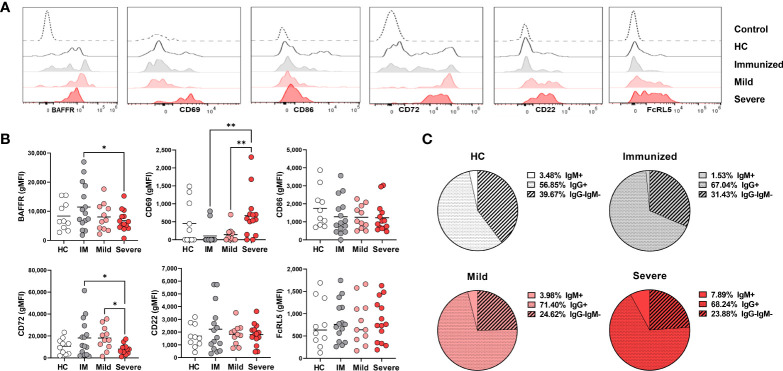
Phenotypic changes in DN2 cells with Severe SARS-CoV-2 infection. **(A)** Representative histograms depicting expression of BAFFR, CD69, CD86, CD72, CD22 and FcRL5 on DN2 cells from a healthy control (HC), individual immunized against SARS-CoV-2 or individuals with mild or severe SARS-CoV-2 infection. Control dotted histogram is an FMO for CD69, CD68, CD72 and CD22. Control dotted histogram for BAFFR and FcRL5 is expression on CD3+ T cells from a healthy control subject. **(B)** Quantification of BAFFR, CD69, CD86, CD72, CD22 and FcRL5 expression (gMFI: geometric mean fluorescent intensity) on DN2 cells from healthy controls (HC), those immunized against SARS-CoV-2 (IM), or individuals with mild or severe SARS-CoV-2 infection. **(C)** Pie charts depicting the average frequency of IgM+, IgG+, or IgG-IgM- DN2 cells for healthy controls (HC, N=10), individuals immunized against SARS-CoV-2 (N=15), and individuals with mild (N=11) or severe SARS-CoV-2 infection (N=14). Statistics: one-way ANOVA, *p < 0.05, **p < 0.01.

Given the increased frequency of DN2 cells with severe SARS-CoV-2 infection (**Figure 1**), we anticipated that these cells might exhibit enhanced survival as indicated by increased expression levels of BAFFR. However, we did not find significant differences in BAFFR expression on DN2 cells from healthy individuals or those with either mild or severe SARS-CoV-2 infection although DN2 cells from severe viral infection trended to have reduced BAFFR expression (**Figures 4A, B**). Notably, immunization against SARS-CoV-2 increased expression levels of BAFFR on DN2 cells (**Figures 4A, B**), suggesting that vaccination may modify longevity of these cells.

To determine how viral infection influenced the activation state of DN2 cells, we quantified levels of the CD69 and CD86 on this subset. DN2 cells from severely infected SARS-CoV-2 subjects had significantly higher levels of CD69 expression, but not CD86, when compared to individuals immunized against SARS-CoV-2 or those with mild infection (**Figures 4A, B**). Together, these data indicate that DN2 cells are more activated with severe infection.

We next again assessed if immunization or viral infection influenced inhibitory receptor expression on DN2 B cells by measuring CD72 and CD22 surface expression. The mean fluorescence intensity (MFI) of CD72 was found to be expressed at a higher level on DN2 cells from immunized individuals and those with mild viral infection compared to healthy controls (**Figures 4A, B**). Accordingly, CD72 was expressed at a significantly lower level on DN2 cells from severe SARS-CoV-2 infection compared to DN2 cells after immunization or those with mild infection (**Figures 4A, B**). Examination of CD22 revealed no significant difference in levels of expression on DN2 cells from each group, however, similar to the CD72 inhibitory receptor, the average CD22 MFI was higher on DN2 cells after immunization against SARS-CoV-2 suggesting that vaccination induces inhibitory receptor expression on DN2 cells (**Figures 4A, B**). DN2 cells from healthy controls and SLE patients were reported to also express the FcRL5 inhibitory receptor (9). Measurement of FcRL5 revealed that there is a wide distribution of expression on DN2 cells in all cohort populations and without significant differences in expression (**Figures 4A, B**). Together, these data indicate that while CD72 expression is depressed with severe SARS-CoV-2 infection, the expression of the CD22 and FCRL5 inhibitory receptors on DN2 cells does not change with severe infection. In contrast, immunization appears to elevate expression of CD22 and CD72 on DN2 B cells.

DN2 cells from healthy controls or individuals with SLE have been reported to predominantly express IgG (9). In accord, our evaluation of immunoglobulin isotype class expressed by DN2 cells reveals that the majority of DN2 cells are IgG^+^, approximately one quarter of DN2 cells are IgG^–^IgM^–^and a smaller fraction are IgM+ (**Figure 4C**). Interestingly, compared to DN2 cells from healthy controls, there was a slight increase in the frequency of IgG^+^ DN2 cells after immunization against SARS-CoV-2, or in individuals with mild or severe SARS-CoV-2 infection (**Figure 4C**). Importantly, with severe SARS-CoV-2 infection we observed a significant increase in IgM^+^ DN2 cells compared to healthy controls and a corresponding decrease in IgG^–^IgM^–^ DN2 cells with severe infection (**Figure 4C**), demonstrating that viral infection alters the composition of immunoglobulin isotype expression in DN2 cells.

Together these data indicate that DN2 cells persist with severe viral infection, are more activated with minimal change in inhibitory receptor expression and slight changes in immunoglobulin isotype class.

### Phenotypic characterization of the novel DN3 cells during severe SARS-CoV-2 infection

**Figure 5:**
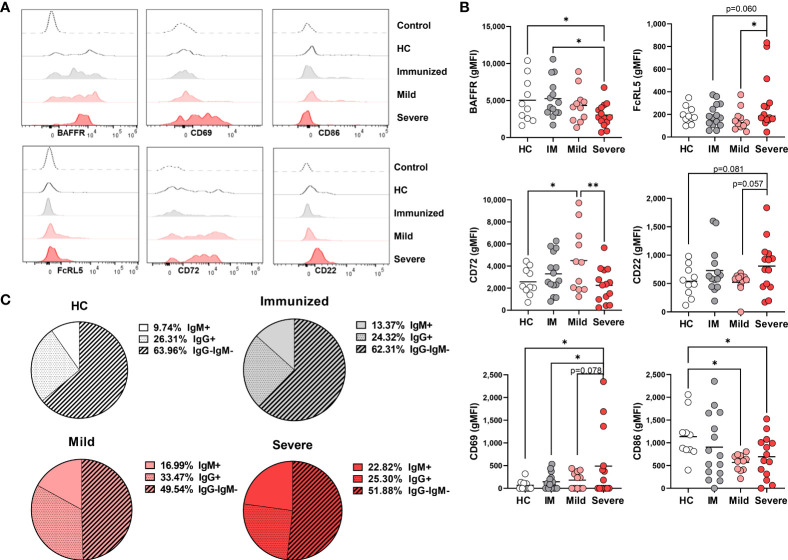
Phenotypic changes in DN3 cells with Severe SARS-CoV-2 infection. **(A)** Representative histograms depicting expression of BAFFR, CD69, CD86, FcRL5, CD72, and CD22 on DN3 cells from a healthy control (HC), a subject immunized against SARS-CoV-2, or individuals with mild or severe SARS-CoV-2 infection. Control dotted histogram is an FMO for CD69, CD68, CD72 and CD22. Control dotted histogram for BAFFR and FcRL5 is expression on CD3+ T cells from a healthy control subject. **(B)** Quantification of BAFFR, CD69, CD86, FcRL5, CD72, and CD22 expression (gMFI: geometric mean fluorescent intensity) on DN3 cells from healthy controls (HC), individuals immunized against SARS-CoV-2 (IM), or individuals with mild or severe SARS-CoV-2 infection. **(C)** Pie charts depicting the average frequency of IgM+, IgG+, or IgG-IgM- DN3 cells for healthy controls (HC, N=10), individuals immunized against SARS-CoV-2 (N=15), and individuals with mild (N=11) or severe SARS-CoV-2 infection (N=14). Statistics: one-way ANOVA, *p < 0.05, **p < 0.01.

DN3 cells are a newly reported subset of the Double Negative population with an unknown phenotype (12). Given the significantly increased frequency of DN3 cells with severe SARS-CoV-2 infection (**Figure 1**), we anticipated that that DN3 cells might display enhanced survival resulting from increased expression levels of BAFFR. Surprisingly, DN3 cells were observed to express significantly lower levels of BAFFR with severe SARS-CoV-2 infection compared to DN3 cells from heathy controls or individuals immunized against SARS-CoV-2 (**Figures 5A, B**). Together these data, along with the maintenance of BAFFR expression on DN2 cells described above (**Figure 4**), suggests the DN2 and DN3 subsets apparently rely less on the survival cytokine BAFF with severe viral infection.

Given that DN2 cells express the FcRL5 inhibitory receptor and expression is unchanged with viral infection (**Figure 4**), we wondered whether DN3 cells also express this receptor. Analysis of FcRL5 expression on DN3 cells reveals that this population appears to normally express low levels of FcRL5 and expression is variably increased on DN3 cells with severe SARS-CoV-2 infection and when compared to immunized individuals or those with mild infection (**Figures 5A, B**). Examination of other inhibitory receptors, CD72 and CD22, revealed that there was a significant increase in expression level of CD72 on DN3 cells from mild SARS-CoV-2 compared to heathy controls, such that DN3 cells from subjects with severe SARS-CoV-2 infection have significantly lower levels of CD72 expression than from mild infection (**Figures 5A, B**). Expression of CD22 on DN3 cells from severe SARS-CoV-2 infection trends to a higher level of expression than DN3 cells from heathy controls and subjects with mild infection (**Figures 5A, B**). Together these data indicate that inhibitory receptor expression on DN3 cells from severe SARS-CoV-2 infection appears modestly increased compared to DN3 cells from healthy controls.

Our analyses of DN1 and DN2 cell subsets show these populations were both highly activated with severe SARS-CoV-2 infection (**Figures 3**, **4**). Therefore, we next asked whether DN3 cells were also activated as indicated by increased expression of the CD69 and CD86 markers. Quantification of CD69 on DN3 cells revealed that, while variable, on average there is a higher level of expression of CD69 on DN3 cells from severe SARS-CoV-2 infection compared to healthy controls, individuals immunized against SARS-CoV-2 and those with mild infection (**Figures 5A, B**). These results suggest severe SARS-CoV-2 infection promotes robust activation on DN3 cells. Intriguingly, expression of CD86 was significantly downregulated on DN3 cells from subjects with mild or severe SARS-CoV-2 infection compared to healthy controls, suggesting DN3 cells may have less capacity to activate T cells with viral infection (**Figures 5A, B**).

To the best of our knowledge, the immunoglobulin isotype class of DN3 cells has not been reported. In this study, the majority of DN3 cells from healthy controls are IgG^–^IgM^–^ (likely IgA given the reported enrichment in *IGHA2* transcripts) (10), followed by an intermediate frequency of IgG^+^ DN3 cells, with a smaller proportion of IgM^+^ DN3 cells (**Figure 5C**). A similar breakdown of IgG^–^IgM^–^ DN3 cells as the most frequent immunoglobulin class followed by an intermediate frequency of IgG^+^ DN3 cells and fewer IgM^+^ DN3 cells was observed in immunized individuals and those with mild or severe SARS-CoV-2 infection (**Figure 5C**). Importantly, with severe SARS-CoV-2 infection we again observed a significant increase in IgM^+^ DN3 cells and a trend to an increase in IgM^+^ DN3 cells with mild infection compared to DN3 cells from healthy controls (**Figure 5C**). These changes were concomitant with significant decreases in the frequency of IgG^–^IgM^–^ DN3 cells from mild or severe SARS-CoV-2 subjects compared to DN3 cells from healthy controls (**Figure 5C**). These data demonstrate that viral infection modifies isotype class dominance in the DN3 subset.

Overall, it is clear that viral infection modulates the phenotype of the novel DN3 subset such that these cells have reduced BAFFR and CD86 but elevated levels of FcRL5, CD22, and CD69 during severe disease. A summary of these findings is depicted in **Supplemental Table 2**.

### Double negative B cells subsets maintain the ability to signal through the BCR during severe SARS-CoV-2 infection

**Figure 6:**
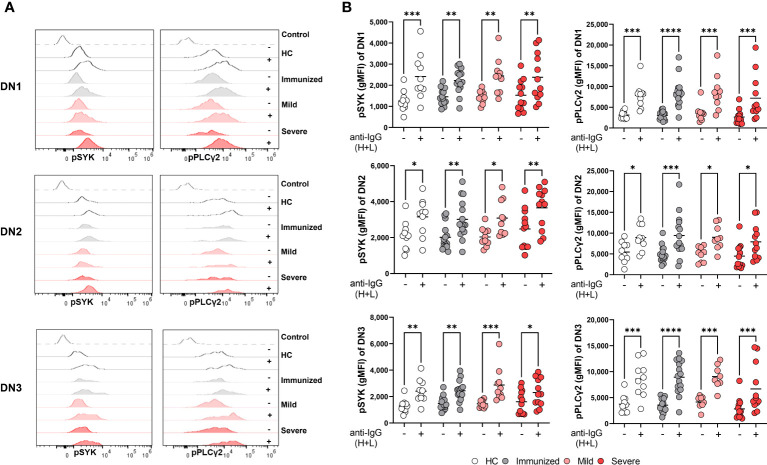
Maintenance of BCR signaling in Double Negative subsets during Severe SARS-CoV-2 infection. **(A)** Representative histograms depicting expression of pSYK and pPLCγ2 on DN1, DN2, or DN3 cells from a healthy control (HC), individual immunized against SARS-CoV-2, and individual with mild or severe SARS-CoV-2 infection without (-) or with (+) stimulation by 10μg/mL anti-IgG (H+L) F(ab’)2 for 5 min. Control dotted histogram is an FMO. **(B)** Quantification of expression levels of pSYK and pPLCγ2 (gMFI: geometric mean fluorescent intensity) on DN1, DN2, or DN3 cells from healthy controls (HC, N=10), individuals immunized against SARS-CoV-2 (N=15), or individuals with mild (N=10) or severe SARS-CoV-2 infection (N=12) without **(-)** or with (+) stimulation by 10μg/mL anti-IgG (H+L) F(ab’)2 for 5 min. Statistics. one-way ANOVA, *p < 0.05, **p < 0.01, ***p < 0.001, ****p < 0.0001.

DN2 cells from SLE patients have the ability to signal through the BCR despite expressing CD22 and FCRL5 inhibitory receptors (9), however the functional capacity of DN2 cells to respond to BCR signaling in healthy individuals or other disease states has not been reported. DN2 cells from severe SARS-CoV-2 infection also express inhibitory receptors and thus we asked whether DN2 cells maintained an ability to signal through the BCR. To address this, PBMCs from healthy controls, immunized individuals, or individuals with either mild or severe SARS-CoV-2 infection were stimulated with anti-human Ig to induce BCR signaling and DN subsets were subsequently assessed for activation of BCR signaling effector molecules as previously described (34). Specifically, we used flow cytometry to measure the mean fluorescence intensity of phospho-SYK (pSYK), a tyrosine kinase signaling molecule proximal to the BCR (34), with and without anti-Ig stimulation. The results from these analyses revealed that DN1 cells from all cohorts (heathy controls, immunized individuals and SARS-CoV-2 mild and severe infected individuals) displayed significant increases in pSYK levels after BCR stimulation (**Figure 6**). Similar increases in pSYK levels were observed for DN2 and DN3 cells in all cohorts (**Figure 6**). We further quantified levels of pPLCγ2, another BCR signaling effector molecule further downstream in the BCR signal transduction cascade. Here, we again found that the B cells within all three DN subsets, DN1, DN2 and DN3, and from each cohort, displayed significant increases in pPLCγ2 levels after BCR stimulation (**Figure 6**). These data indicate that despite changes in B cell inhibitory receptor expression with severe SARS-CoV-2 infection observed above, the B cells within all DN subsets maintain the ability to transduce signals originating from the BCR.

**Figure 7:**
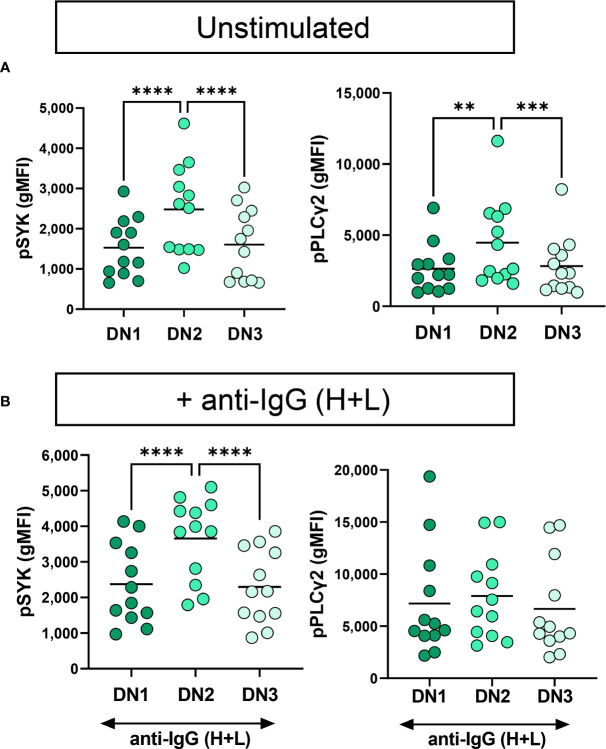
Functional comparison of DN1, DN2 and DN3 cells in severe SARS-CoV-2 infection. **(A)** Quantification of expression level of pSYK or pPLCγ2 on DN1, DN2 or DN3 cells from individuals with severe SARS-CoV-2 infection (N=12) without (unstimulated) or **(B)** with stimulation by 10μg/mL anti-IgG (H+L) F(ab’)2 for 5 min. Statistics. one-way ANOVA, **p < 0.01, ***p < 0.001, ****p < 0.0001.

We have previously reported that severe SARS-CoV-2 infection enhances BCR signaling by B_ND_ cells, a typically functionally anergic B cell population in healthy controls (24). To determine if DN2 or any of the B cells within the different DN subsets display enhanced BCR signaling with severe viral infection, we calculated the fold difference of pSYK and pPLγC2 between unstimulated and BCR-stimulated B cells and compared these values between cohorts. The results of these analyses did not reveal any significant enhancement in the ability of B cells from any DN subset, or cohort, to transduce BCR signals leading to SYK and PLCγ2 activation (**Supplemental Figure 1**). Thus, despite residing at a higher activation level as observed above (CD69), DN2 cells do not display an enhancement in BCR signaling with severe viral infection. However, we noted that DN2 cells from healthy controls, immunized individuals and individuals with mild infection displayed increased basal levels of pSYK and pPLCγ2 relative to DN1 and DN3 cells (**Supplemental Figure 2**). We observed a similar higher basal level of pSYK and pPLCγ2 expression in DN2 cells compared to DN1 or DN3 cells from individuals with severe SARS-CoV-2 infection (**Figure 7**). This suggests that DN2 cells naturally have the largest capacity to signal through the BCR compared to other DN subsets, a process which may be independent of traditional inhibitory receptors (CD22, CD72, FcRL5) and/or that the function of inhibitory receptors is impaired during viral infection.

### Systemic inflammation during severe SARS-CoV-2 infection is linked to alteration in Double NegativeB cell frequency

**Figure 8:**
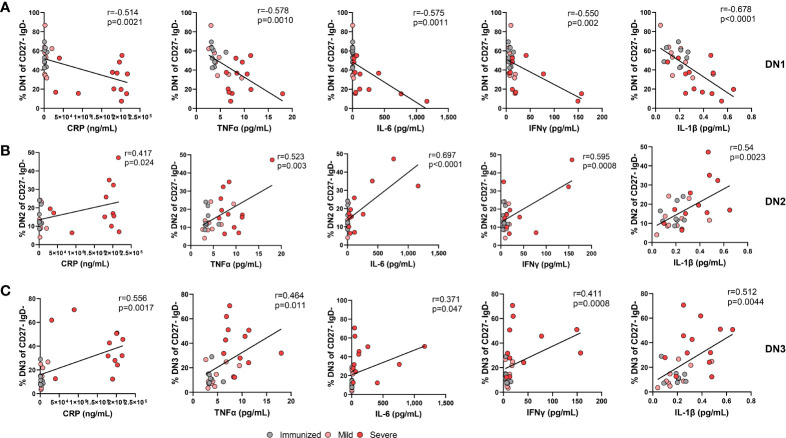
The alteration in the frequency of Double Negative populations correlates with increased systemic inflammation during Severe SARS-CoV-2 infection. **(A)** Correlation of systemic cytokine levels (CRP, TNFα, IL-6, IFNγ, IL-1β) in individuals immunized against SARS-CoV-2 (grey circles, N=8), individuals with mild (pink circle, N=9) and severe SARS-CoV-2 infection (red circle, N=12) with frequency of DN1 cells, **(B)** frequency of DN2 cells, or **(C)** frequency of DN3 cells within total double negative population (CD27-IgD-). Statistics: Pearson correlation, r and p values noted on each panel.

Inflammatory cytokines such as IFNλ and IFNγ are able to promote the *in vitro* generation of DN2 cells from naive B cells (9, 14, 15) and we and others have reported an increase in pro-inflammatory cytokines with severe SARS-CoV-2 infection (**Supplemental Table 1**) (16, 24, 35). Accordingly, we questioned whether the increased systemic inflammation associated with severe viral infection might account for the observed changes in DN subset frequencies. We began to address this by measuring levels of C-reactive protein (CRP) in plasma, an indicator of overall inflammatory state, from immunized and virally infected cohorts and as expected found that CRP levels were significantly higher in severe SARS-CoV-2 when compared to levels found in individuals immunized against SARS-CoV-2 (**Supplemental Table 1**). Furthermore, levels of CRP significantly correlated negatively with the frequency of DN1 cells within the Double Negative population (**Figure 8A**). In line with the CRP correlation, there were also significant negative correlations between the levels of TNF, IL-6, IFNγ, and IL-1β and the frequency of DN1 cells (**Figure 8A**). These data indicate that SARS-CoV-2 associated inflammation promotes a loss of the DN1 population. In contrast, the observed increases in the DN2 subset were significantly and positively correlated with levels of CRP as was the frequency of DN2 cells within the Double Negative population (**Figure 8B**), in keeping with similar previous observations (12, 21). We further observed significant positive correlations between the levels of specific cytokines including TNF, IL-6, IFNγ, and IL-1β with the increased frequency of DN2 cells (**Figure 8B**). These data suggest that the inflammation associated with severe SARS-CoV-2 infection likely drives expansion of the DN2 subset. Moreover, correlation of levels of CRP with the frequency of DN3 cells again demonstrated a significant positive relationship (**Figure 8C**), and as previously reported (11). There were again significant positive correlations between the levels of the TNF, IL-6, IFNγ, and IL-1β proinflammatory cytokines and the frequency of DN3 cells in our cohorts (**Figure 8C**). Overall, these data link broad systemic inflammation and specific inflammatory cytokines with the observed changes in specific DN subset frequencies during severe SARS-CoV-2 infection.

### Changes in Double Negative B cell frequency are linked to increased levels of autoreactive antibodies during severe SARS-CoV-2 infection

**Figure 9:**
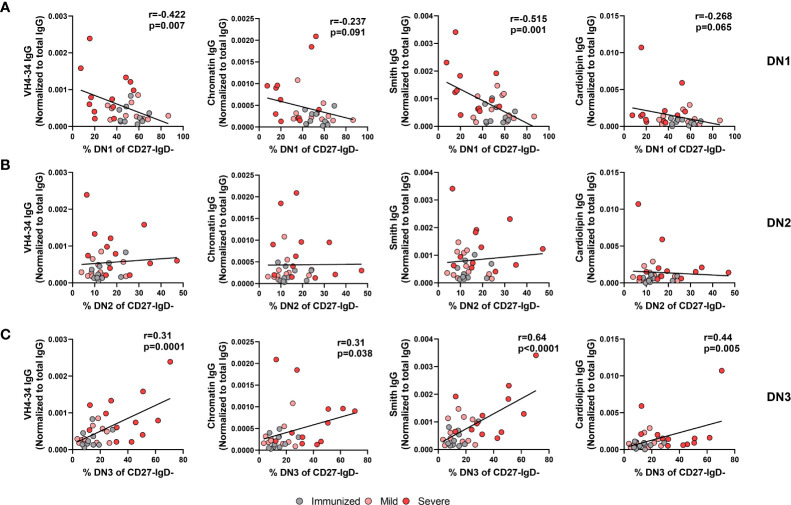
Correlation of autoreactive antibodies with changes in Double Negative subset frequencies during Severe SARS-CoV-2 infection. **(A)** Correlation of frequency of DN1 cells, **(B)** frequency of DN2 cells, or **(C)** frequency of DN3 cells within total double negative population (CD27-IgD-) with systemic levels of autoreactive IgG antibodies (VH4-34, Chromatin, Smith, cardiolipin) normalized to total IgG from individuals immunized against SARS-CoV-2 (N=10), and individuals with mild (N=11) or severe SARS-CoV-2 infection (N=13). Statistics: Pearson correlation, r and p values noted on panel.

We and others have reported an increase in systemic autoreactive antibodies during severe SARS-CoV-2 infection (**Supplemental Table 1**) (18–20, 24, 36), suggesting an infection-induced breach in immunological tolerance and expansion of autoreactive B cells in response to inflammation during viral infection. Whereas DN1 cells are not thought to harbor autoreactive specificities, DN2 cells were demonstrated to be enriched in cells harboring a VH4-34 specificity (9), an indicator of autoreactivity in SLE patients (26, 37). Expression of germline VH4-34 Ig heavy chain in human B cells is associated with BCRs with specificity to nuclear antigen, chromatin, CD45 and glycoproteins on red blood cells (26, 37). We and others have demonstrated that serum autoantibodies of VH4-34 origin are elevated in severe SARS-CoV-2 infection (12, 24). The specificities displayed by DN3 cells, and whether there is a similar enrichment in autoreactive specificity, has not been established. As we observe increased frequencies of both DN2 and DN3 subsets with severe viral infection, we asked whether either DN2 or DN3 subset was associated with autoantibody production during severe SARS-CoV-2 infection. Correlating of the frequency of DN1 cells within the Double Negative population with the plasma titer of VH4-34 IgG normalized to total IgG levels revealed a significant negative correlation between these parameters (**Figure 9A**). Similarly, we observed a significant negative correlation between the frequency of DN1 cells and the relative titer of anti-Smith IgG antibodies (**Figure 9A**). A trend was further observed between the correlation of the frequency of DN1 cells with the relative titers of anti-Chromatin IgG or anti-Cardiolipin IgG (**Figure 9A**). These data indicate that a reduction in the presence of DN1 cells with severe viral infection correlates with a rise in autoreactive antibody titers suggesting that DN1 cells may be limiting in the formation of autoantibodies.

Surprisingly, despite the reported enrichment of autoreactive specificities within the DN2 cell subset in SLE, our data did not demonstrate any significant correlation between the frequency of DN2 cells within the Double Negative population and the relative titers of VH4-34, anti-Chromatin, anti-Smith or anti-cardiolipin IgG (**Figure 9B**). In contrast, we instead find that a significant positive correlation exists between the frequency of DN3 cells within the Double Negative population and relative titers of VH4-34 (**Figure 9C**). Furthermore, the frequency of DN3 cells significantly and positively correlated with the relative titers of not only VH4-34 but also anti-chromatin IgG, anti-Smith IgG, and anti-cardiolipin IgG (**Figure 9C**). Importantly, there was a significant negative correlation between the frequency of DN3 and DN1 cells, whereas no correlation was observed between the frequency of DN3 and DN2 cells (**Supplemental Figure 3**). These data suggest that as DN3 cells expand in frequency with severe SARS-CoV-2 infection so too do the levels of autoreactive antibodies and implicating this novel cell type as a potential driver of autoimmunity. Together these data indicate a strong link between the alteration in the frequency of DN1 and DN3 subsets during severe SARS-CoV-2 infection and the development of autoreactive antibodies.

## Discussion

In this report we have interrogated the proportion, phenotype, and functional ability of DN1, DN2 and DN3 B cell subsets in healthy individuals, individuals immunized against SARS-CoV-2 and those with either mild or severe SARS-CoV-2 infection. We also quantified the level of inflammation by measurement of C-reactive protein and specific proinflammatory cytokines in plasma as well as titers of specific serum autoantibodies. From these findings we confirm a striking alteration in composition of the Double Negative population in individuals with severe SARS-CoV-2 infection such that the frequency of DN1 B cells decreases, whereas the DN2 and DN3 subset frequencies increase. We reveal by in-depth characterization of the DN subsets that B cells within each DN1, DN2, and DN3 subset with severe viral infection are highly activated, express altered frequencies of immunoglobulin isotype class, and maintain ability to signal *via* the BCR, suggesting these DN B cells are able to mount an antibody response. Importantly, an increased frequency of DN3 cells with concomitant loss of DN1 cells significantly correlated with increased systemic inflammation and autoreactive antibody production during SARS-CoV-2 infection. Thus, these data provide strong evidence that inflammatory cytokines promote alteration of the Double Negative B cell compartment during SARS-CoV-2 infection, likely contributing to the production of autoreactive antibodies with viral infection.

We present data in this study suggesting that the expansion of the DN2 and DN3 subsets and contraction of the DN1 subset in severe SARS-CoV-2 infection is driven by elevated virally-associated cytokines. Expansion of DN2 in SLE is associated with elevated levels of IFN-γ or IFN-λ (9, 14, 15). Importantly, exposure of naive B cells *in vitro* to IFN-γ or IFN-λ in the presence of TLR7L, IL-21, BAFF and BCR stimulation promotes generation of DN2 cells (9, 15, 38), demonstrating a role for inflammatory cytokines and viral ligands in driving expansion of the DN2 subset. Given these reports, and that we and others have reported increased levels of IFN-γ with SARS-CoV-2 infection (16, 24, 39), it is possible that an analogous process occurs *in vivo* during severe viral infection to promote DN2 subset expansion. As the DN3 population has only recently been identified, the B cell developmental pathway(s) leading to a DN3 cell is not clear, although trajectory analysis suggests that DN3 cells may be precursors to DN2 cells (10); in this study we confirm previous findings that elevated levels of CRP in severe SARS-CoV-2 infection is associated with an increased frequency of DN3 cells (11). Further supporting the role for viral-associated inflammation in driving expansion of the DN3 subset, we identify four specific pro-inflammatory cytokines (IFN-γ, TNF-α, IL-6, and IL-1β) that positively correlate with increased DN3 frequencies. To the best of our knowledge, no other studies have reported an expansion of DN2 or DN3 cells with other viruses. One study did report an expansion of the total DN population after immunization with live attenuated virus against tick borne encephalitis, however the exact subset(s) responsible for the increased frequency was not determined (6). Based on our data here we speculate that if systemic inflammation increases with viral infection or vaccination with whole virus so too would expansion of the DN2 and DN3 subsets. The precise mechanism(s) by which inflammatory cytokines modulate the expansion of DN3/DN2 cells and loss of DN1 cells during severe SARS-CoV-2 infection is an area of current investigation.

Here in this report, we have confirmed the expansion of DN2 and DN3 subsets associated with severe SARS-CoV-2 infection reported by others (11, 21, 22). It remains to be determined whether these subsets contribute to protective humoral responses during viral infection or contribute to COVID-19 disease pathology or both. In severe SARS-CoV-2 infection, broad immune activation of the extrafollicular B cell pathway (a dominant pathway for DN2 generation in SLE) was associated with poor clinical outcomes (12) and an elevated presence of DN3 cells has been associated with worse clinical outcomes (23), suggesting pathogenic roles for DN2 and DN3 cells. Importantly, DN2 cells are enriched in autoreactive VH4-34 clones in SLE and are precursors to autoreactive antibody secreting cells (9), and we and others have reported an increase in VH4-34 IgG levels during severe SARS-CoV-2 infection (12, 24). In this study we demonstrate that expansion of the DN3 subset positively correlates with increased levels of the specific autoreactive antibodies we tested (VH4-34, chromatin, Smith, cardiolipin) indicating that DN3 cells may contribute to autoantibody production during SARS-CoV-2 infection. Conversely, SARS-CoV-2 DN2 and DN3 cells able to bind Spike Receptor Binding Domain (RBD) have been identified, suggesting that viral infection can induce expansion of B cells with specificity to neutralizing epitopes in the Double Negative population (21, 22), although whether DN subsets have specificity to other viral antigens remains to be determined. Interestingly, a preprint study has identified an antibody with specificity to both SARS-CoV-2 and glomerular basement membrane, indicating that cross-reactive antibodies that are anti-viral and autoreactive can be produced during SARS-CoV-2 infection (Woodruff et al., 2021 medRxiv). Finally, determining if DN3 cells are a key source of autoantibodies and/or contribute to pathology observed in severe COVID-19 is an area of active investigation.

We did not address whether the expansion of the DN2 and DN3 subsets with severe viral infection was transient or durable. Our data here on the presence of these DN subsets in healthy controls, immunized individuals and those with mild viral infection would suggest that with resolution of viral-associated inflammation, the frequency of DN2 and DN3 subsets will revert back to levels that are observed in healthy controls. In support of this, a decreased frequency of RBD-specific DN2 cells was observed 10 weeks post-infection when compared to levels during acute infection (22), suggesting that expansion of virus-specific DN2 cells is transient and resolves with infection. However, it should be noted that even five months post-recovery from severe infection, RBD-specific Double Negative B cells are still detectable (40), indicating that expansion of virally-associated DN subsets likely contracts with infection but are not lost. Furthermore, expansion of atypical memory B cells (CD27^–^CD21^lo/–^), which would include both DN2 and DN3 subsets, is resolved in patients recovered from severe SARS-CoV-2 infection (41, 42). Importantly, 6 months post-recovery from severe SARS-CoV-2 infection, one group reports a reduction in autoreactive antibody levels, suggesting a loss or contraction of the autoreactive B cell population (Woodruff et al., 2021 medRxiv). It would be of interest to determine if those who present to the clinic as COVID-19 ‘long-haulers’ maintain expansion of DN2 and DN3 subsets to determine if these subsets play a role in remaining disease pathology.

Previous studies of atypical memory B cells (CD27^–^CD21^lo/–^), that by definition include DN2 and DN3 cells, in the context of malaria and HIV viral infection have reported these B cells to display impaired BCR signaling and proliferative responses (43, 44), a finding that has also been demonstrated in atypical memory B cells from patients with rheumatoid arthritis and Common Variable Immunodeficiency (45, 46). In this study, we do not find any evidence of defective BCR signaling in the DN1, DN2 or DN3 cells from healthy individuals or those with severe SARS-CoV-2 infection. In support of our findings, it has been shown that DN2 cells from SLE patients display intact BCR signaling as determined by levels of the BCR signaling effector, phospho-BLNK (9). Interestingly, we show that DN2 cells from severe SARS-CoV-2 infection had the highest level of pSYK expression upon BCR stimulation when compared with DN1 or DN3 cells. Overall, these findings indicate that DN1, DN2 or DN3 cells have functional BCR responses during severe SARS-CoV-2 infection. The evaluation of CD86 expression on DN subsets from healthy individuals and individuals with severe SARS-CoV-2 infection is also potentially informative given that CD86 provides a costimulatory signal to T cells. In healthy individuals, CD86 expression on DN1 cells is relatively low and compared to DN2 and DN3 suggesting that these latter populations in healthy individuals are poised to interact with T cells. In contrast, with severe viral infection, DN3 cells lose CD86 expression possibly indicating these cells may be less likely to receive T cell help.

There are limited studies examining immunoglobulin gene family usage, specifically in the Double Negative B cell subsets, during SARS-CoV-2 infection. Analysis of the V(D)J repertoire in antibody secreting cells from severe SARS-CoV-2 infection demonstrated significant oligoclonal expansion with low mutation frequencies (majority expressing germline VH genes) and autoreactive VH4-34 gene usage (12). This pattern has been associated with extrafollicular B cell activation (38), particularly in the DN2 subset during an SLE flare (9). Here in this study we demonstrate that increased frequency of DN3 cells positively correlates with the increase in VH4-34 IgG autoreactive antibodies, suggesting DN3 may be a source of autoreactive antibodies and that DN3 cells do not edit against autoreactive VH4-34 gene family usage. However, more work is needed to determine the degree of somatic hypermutation away from germline and if there is preferential immunoglobulin heavy chain family gene usage in each DN subset, including the novel DN3 cells, during viral infection.

In summary, our study provides evidence that the human Double Negative B cell compartment is altered during severe SARS-CoV-2 infection and that this change in subset type significantly correlates with inflammation and production of autoreactive antibodies. Importantly, these findings also imply that an additional B cell subset, the novel DN3 cells, may contribute to autoreactive antibody production. It remains to be determined whether these autoreactive antibodies are also cross-reactive and can mediate an anti-viral response during infection.

## Data availability statement

The raw data supporting the conclusions of this article will be made available by the authors, without undue reservation.

## Ethics statement

The studies involving human participants were reviewed and approved by The Colorado Multiple Institutional Review Board (COMIRB) at the University of Colorado School of Medicine and National Jewish Health approved the use of human plasma and PBMCs, and this study was performed under the Declaration of Helsinki. The patients/participants provided their written informed consent to participate in this study.

## Author contributions

MC and RT designed the study, interpreted the data and wrote the manuscript. MC recruited immunized and mild SARS-CoV-2 subjects, performed experiments, and analyzed the data. MMS and NT performed experiments. MJS and RP provided expertise and samples and helped interpret results. KL, BP, JM, KM, WJ and JB provided mild or severe SARS-CoV-2 samples. All authors reviewed the manuscript. All authors contributed to the article and approved the submitted version.

## Funding

The work was supported by the National Institute of Health Grant R01 AI136534 (RT), AI124474 (RP) and AI131639 (RP). The CU Anschutz CTRC/CCTSI collected whole blood and was supported by NIH/NCATS Colorado CTSA Grant Number UL1 TR002535.

## Acknowledgments

We appreciate those who donated blood for this study to be conducted. We acknowledge the ImmunoMicro Flow Cytometry Shared Resource Laboratory at the University of Colorado Anschutz Medical Campus (RRID : SCR_021321), Christine Griesmer, Ayal Levi, Jazalle McClendon, and Shannon McManus for enrolling patients with severe SARS-CoV-2 and processing the biological specimens. We appreciate helpful discussions with Sophie Hillion Weber from University of Brest.

## Conflict of interest

The authors declare that the research was conducted in the absence of any commercial or financial relationships that could be construed as a potential conflict of interest.

## Publisher’s note

All claims expressed in this article are solely those of the authors and do not necessarily represent those of their affiliated organizations, or those of the publisher, the editors and the reviewers. Any product that may be evaluated in this article, or claim that may be made by its manufacturer, is not guaranteed or endorsed by the publisher.

## References

[1] Kaminski DA Wei C Qian Y Rosenberg AF Sanz I. Advances in human b cell phenotypic profiling. Front Immunol (2012) 3:302. doi: 10.3389/fimmu.2012.00302 23087687 PMC3467643

[2] Sanz I Wei C Jenks SA Cashman KS Tipton C Woodruff MC. Challenges and opportunities for consistent classification of human b cell and plasma cell populations. Front Immunol (2019) 10:2458. doi: 10.3389/fimmu.2019.02458 31681331 PMC6813733

[3] Huang W Sinha J Newman J Reddy B Budhai L Furie R. The effect of anti-CD40 ligand antibody on b cells in human systemic lupus erythematosus. Arthritis rheumatism (2002) 46(6):1554–62. doi: 10.1002/art.10273 12115186

[4] Anolik JH Barnard J Cappione A Pugh-Bernard AE Felgar RE Looney RJ. Rituximab improves peripheral b cell abnormalities in human systemic lupus erythematosus. Arthritis rheumatism (2004) 50(11):3580–90. doi: 10.1002/art.20592 15529346

[5] Wei C Anolik J Cappione A Zheng B Pugh-Bernard A Brooks J. A new population of cells lacking expression of CD27 represents a notable component of the b cell memory compartment in systemic lupus erythematosus. J Immunol (2007) 178(10):6624–33. doi: 10.4049/jimmunol.178.10.6624 17475894

[6] Ruschil C Gabernet G Lepennetier G Heumos S Kaminski M Hracsko Z. Specific induction of double negative b cells during protective and pathogenic immune responses. Front Immunol (2020) 11:606338. doi: 10.3389/fimmu.2020.606338 33391273 PMC7775384

[7] Fraussen J Marquez S Takata K Beckers L Montes Diaz G Zografou C. Phenotypic and ig repertoire analyses indicate a common origin of IgD(-)CD27(-) double negative b cells in healthy individuals and multiple sclerosis patients. J Immunol (2019) 203(6):1650–64. doi: 10.4049/jimmunol.1801236 PMC6736705 31391234

[8] Richardson CT Slack MA Dhillon G Marcus CZ Barnard J Palanichamy A. Failure of b cell tolerance in CVID. Front Immunol (2019) 10:2881. doi: 10.3389/fimmu.2019.02881 31921145 PMC6914825

[9] Jenks SA Cashman KS Zumaquero E Marigorta UM Patel AV Wang X. Distinct effector b cells induced by unregulated toll-like receptor 7 contribute to pathogenic responses in systemic lupus erythematosus. Immunity (2018) 49(4):725–39.e6. doi: 10.1016/j.immuni.2018.08.015 30314758 PMC6217820

[10] Stewart A Ng JC Wallis G Tsioligka V Fraternali F Dunn-Walters DK. Single-cell transcriptomic analyses define distinct peripheral b cell subsets and discrete development pathways. Front Immunol (2021) 12:602539. doi: 10.3389/fimmu.2021.602539 33815362 PMC8012727

[11] Sosa-Hernandez VA Torres-Ruiz J Cervantes-Diaz R Romero-Ramirez S Paez-Franco JC Meza-Sanchez DE. B cell subsets as severity-associated signatures in COVID-19 patients. Front Immunol (2020) 11:611004. doi: 10.3389/fimmu.2020.611004 33343585 PMC7744304

[12] Woodruff MC Ramonell RP Nguyen DC Cashman KS Saini AS Haddad NS. Extrafollicular b cell responses correlate with neutralizing antibodies and morbidity in COVID-19. Nat Immunol (2020) 21(12):1506–16. doi: 10.1038/s41590-020-00814-z PMC7739702 33028979

[13] Jenks SA Wei C Bugrovsky R Hill A Wang X Rossi FM. B cell subset composition segments clinically and serologically distinct groups in chronic cutaneous lupus erythematosus. Ann rheumatic dis (2021) 80(9):1190–200. doi: 10.1136/annrheumdis-2021-220349 PMC8906255 34083207

[14] Zumaquero E Stone SL Scharer CD Jenks SA Nellore A Mousseau B. IFNgamma induces epigenetic programming of human T-bet(hi) b cells and promotes TLR7/8 and IL-21 induced differentiation. eLife (2019) 8:1–36. doi: 10.7554/eLife.41641 PMC6544433 31090539

[15] Barnas JL Albrecht J Meednu N Alzamareh DF Baker C McDavid A. B cell activation and plasma cell differentiation are promoted by IFN-lambda in systemic lupus erythematosus. J Immunol (2021) 207(11):2660–72. doi: 10.4049/jimmunol.2100339 PMC8612983 34706932

[16] Yang L Liu S Liu J Zhang Z Wan X Huang B. COVID-19: immunopathogenesis and immunotherapeutics. Signal transduct targeted Ther (2020) 5(1):128. doi: 10.1038/s41392-020-00243-2 PMC7381863 32712629

[17] Wang EY Mao T Klein J Dai Y Huck JD Liu F. Diverse functional autoantibodies in patients with COVID-19. medRxiv: Preprint server Health Sci (2021) 595:283–288. doi: 10.1101/2020.12.10.20247205 PMC13130511 34010947

[18] Bastard P Rosen LB Zhang Q Michailidis E Hoffmann HH Zhang Y. Autoantibodies against type I IFNs in patients with life-threatening COVID-19. Science (2020) 370(6515):1–12. doi: 10.1126/science.abd4585 PMC7857397 32972996

[19] Chang SE Feng A Meng W Apostolidis SA Mack E Artandi M. New-onset IgG autoantibodies in hospitalized patients with COVID-19. Nat Commun (2021) 12(1):5417. doi: 10.1038/s41467-021-25509-3 34521836 PMC8440763

[20] Xiao M Zhang Y Zhang S Qin X Xia P Cao W. Antiphospholipid antibodies in critically ill patients with COVID-19. Arthritis Rheumatol (2020) 72(12):1998–2004. doi: 10.1002/art.41425 32602200 PMC7361932

[21] Kaneko N Kuo HH Boucau J Farmer JR Allard-Chamard H Mahajan VS. Loss of bcl-6-Expressing T follicular helper cells and germinal centers in COVID-19. Cell (2020) 183(1):143–57.e13. doi: 10.1016/j.cell.2020.08.025 32877699 PMC7437499

[22] de Campos-Mata L Tejedor Vaquero S Tacho-Pinot R Pinero J Grasset EK Arrieta Aldea I. SARS-CoV-2 sculpts the immune system to induce sustained virus-specific naive-like and memory b-cell responses. Clin Trans Immunol (2021) 10(9):e1339. doi: 10.1002/cti2.1339 PMC8418925 34504693

[23] Cervantes-Diaz R Sosa-Hernandez VA Torres-Ruiz J Romero-Ramirez S Canez-Hernandez M Perez-Fragoso A. Severity of SARS-CoV-2 infection is linked to double-negative (CD27(-) IgD(-)) b cell subset numbers. Inflammation Res (2022) 71(1):131–40. doi: 10.1007/s00011-021-01525-3 PMC8631699 34850243

[24] Castleman MJ Stumpf MM Therrien NR Smith MJ Lesteberg KE Palmer BE. SARS-CoV-2 infection relaxes peripheral b cell tolerance. J Exp Med (2022) 219(6):1–15. doi: 10.1084/jem.20212553 PMC9014793 35420627

[25] Isenberg D Spellerberg M Williams W Griffiths M Stevenson F. Identification of the 9G4 idiotope in systemic lupus erythematosus. Br J Rheumatol (1993) 32(10):876–82. doi: 10.1093/rheumatology/32.10.876 7691367

[26] Richardson C Chida AS Adlowitz D Silver L Fox E Jenks SA. Molecular basis of 9G4 b cell autoreactivity in human systemic lupus erythematosus. J Immunol (2013) 191(10):4926–39. doi: 10.4049/jimmunol.1202263 PMC3816606 24108696

[27] Lenschow DJ Sperling AI Cooke MP Freeman G Rhee L Decker DC. Differential up-regulation of the B7-1 and B7-2 costimulatory molecules after ig receptor engagement by antigen. J Immunol (1994) 153(5):1990–7. 7519638

[28] Jenks SA Cashman KS Woodruff MC Lee FE Sanz I. Extrafollicular responses in humans and SLE. Immunol Rev (2019) 288(1):136–48. doi: 10.1111/imr.12741 PMC6422038 30874345

[29] Smulski CR Eibel H. BAFF and BAFF-receptor in b cell selection and survival. Front Immunol (2018) 9:2285. doi: 10.3389/fimmu.2018.02285 30349534 PMC6186824

[30] Smith KG Tarlinton DM Doody GM Hibbs ML Fearon DT. Inhibition of the b cell by CD22: a requirement for Lyn. J Exp Med (1998) 187(5):807–11. doi: 10.1084/jem.187.5.807 PMC2212179 9480991

[31] Adachi T Wakabayashi C Nakayama T Yakura H Tsubata T. CD72 negatively regulates signaling through the antigen receptor of b cells. J Immunol (2000) 164(3):1223–9. doi: 10.4049/jimmunol.164.3.1223 10640734

[32] Haga CL Ehrhardt GR Boohaker RJ Davis RS Cooper MD. Fc receptor-like 5 inhibits b cell activation *via* SHP-1 tyrosine phosphatase recruitment. Proc Natl Acad Sci United States America (2007) 104(23):9770–5. doi: 10.1073/pnas.0703354104 PMC1887609 17522256

[33] Franks SE Cambier JC. Putting on the brakes: Regulatory kinases and phosphatases maintaining b cell anergy. Front Immunol (2018) 9:665. doi: 10.3389/fimmu.2018.00665 29681901 PMC5897502

[34] Packard TA Cambier JC. B lymphocyte antigen receptor signaling: initiation, amplification, and regulation. F1000prime Rep (2013) 5:40. doi: 10.12703/P5-40 24167721 PMC3790562

[35] Darif D Hammi I Kihel A El Idrissi Saik I Guessous F Akarid K. The pro-inflammatory cytokines in COVID-19 pathogenesis: What goes wrong? Microbial pathogenesis (2021), 153:104799. doi: 10.1016/j.micpath.2021.104799 33609650 PMC7889464

[36] Wang EY Mao T Klein J Dai Y Huck JD Jaycox JR. Diverse functional autoantibodies in patients with COVID-19. Nature. (2021) 595(7866):283–8. doi: 10.1038/s41586-021-03631-y PMC13130511 34010947

[37] Cappione AJ Pugh-Bernard AE Anolik JH Sanz I. Lupus IgG VH4.34 antibodies bind to a 220-kDa glycoform of CD45/B220 on the surface of human b lymphocytes. J Immunol (2004) 172(7):4298–307. doi: 10.4049/jimmunol.172.7.4298 15034044

[38] Tipton CM Fucile CF Darce J Chida A Ichikawa T Gregoretti I. Diversity, cellular origin and autoreactivity of antibody-secreting cell population expansions in acute systemic lupus erythematosus. Nat Immunol (2015) 16(7):755–65. doi: 10.1038/ni.3175 PMC4512288 26006014

[39] Del Valle DM Kim-Schulze S Huang HH Beckmann ND Nirenberg S Wang B. An inflammatory cytokine signature predicts COVID-19 severity and survival. Nat Med (2020) 26(10):1636–43. doi: 10.1038/s41591-020-1051-9 PMC7869028 32839624

[40] Reyes RA Clarke K Gonzales SJ Cantwell AM Garza R Catano G. SARS-CoV-2 spike-specific memory b cells express higher levels of T-bet and FcRL5 after non-severe COVID-19 as compared to severe disease. PloS One (2021) 16(12):e0261656. doi: 10.1371/journal.pone.0261656 34936684 PMC8694470

[41] Oliviero B Varchetta S Mele D Mantovani S Cerino A Perotti CG. Expansion of atypical memory b cells is a prominent feature of COVID-19. Cell Mol Immunol (2020) 17(10):1101–3. doi: 10.1038/s41423-020-00542-2 PMC7463104 32879471

[42] Wildner NH Ahmadi P Schulte S Brauneck F Kohsar M Lutgehetmann M. B cell analysis in SARS-CoV-2 versus malaria: Increased frequencies of plasmablasts and atypical memory b cells in COVID-19. J leukoc Biol (2021) 109(1):77–90. doi: 10.1002/JLB.5COVA0620-370RR 33617048 PMC10016889

[43] Moir S Ho J Malaspina A Wang W DiPoto AC O’Shea MA. Evidence for HIV-associated b cell exhaustion in a dysfunctional memory b cell compartment in HIV-infected viremic individuals. J Exp Med (2008) 205(8):1797–805. doi: 10.1084/jem.20072683 PMC2525604 18625747

[44] Portugal S Tipton CM Sohn H Kone Y Wang J Li S. Malaria-associated atypical memory b cells exhibit markedly reduced b cell receptor signaling and effector function. eLife (2015:1–21) 4. doi: 10.7554/eLife.07218 PMC4444601 25955968

[45] Rakhmanov M Keller B Gutenberger S Foerster C Hoenig M Driessen G. Circulating CD21low b cells in common variable immunodeficiency resemble tissue homing, innate-like b cells. Proc Natl Acad Sci United States America (2009) 106(32):13451–6. doi: 10.1073/pnas.0901984106 PMC2726348 19666505

[46] Isnardi I Ng YS Menard L Meyers G Saadoun D Srdanovic I. Complement receptor 2/CD21- human naive b cells contain mostly autoreactive unresponsive clones. Blood. (2010) 115(24):5026–36. doi: 10.1182/blood-2009-09-243071 PMC3373152 20231422

