# Supplementary material for: Autoantibodies elicited with SARS-CoV-2 infection are linked to alterations in double negative B cells
Source: Front Immunol. 2022 Sep 5;13:988125. doi: 10.3389/fimmu.2022.988125 (PMC9484582; doi:10.3389/fimmu.2022.988125)
Supplement: Supplementary file 1 [file DataSheet_1.docx]

**Supplemental Materials and Methods for**

**“Autoantibodies Elicited with SARS-CoV-2 infection are Linked to Alterations in Double Negative B cells”**

Moriah J. Castleman,^1^ Megan M. Stumpf,^1^ Nicholas R. Therrien,^1^ Mia J. Smith,^1,2^ Kelsey E. Lesteberg, ^1,3^ Brent E. Palmer,^4^ James P. Maloney, ^5^ William J. Janssen,^6,7^ Kara J. Mould,^6,7^ J. David Beckham,^1,3,8^ Roberta Pelanda,^1^ Raul M. Torres^1*^

^1^ Department of Immunology and Microbiology, University of Colorado School of Medicine, Aurora, Colorado, USA.

^2^ Barbara Davis Center for Diabetes, Department of Pediatrics, University of Colorado School of Medicine, Aurora, Colorado, USA.

^3^ Department of Medicine, Division of Infectious Disease, University of Colorado School of Medicine, Aurora, Colorado, USA.

^4^ Department of Medicine, Division of Allergy and Clinical Immunology, University of Colorado School of Medicine, Aurora, Colorado, USA.

^5^ Department of Medicine, Division of Pulmonary Sciences and Critical Care Medicine, University of Colorado School of Medicine, Aurora, Colorado, USA.

^6^ Department of Medicine, National Jewish Health, Denver, Colorado, USA.

^7^ Department of Medicine, University of Colorado, Aurora, Colorado, USA.

^8^ Rocky Mountain Regional VA, Medical Center, Aurora, Colorado, USA.

*Corresponding author: Raul Torres, Raul.Torres@cuanschutz.edu


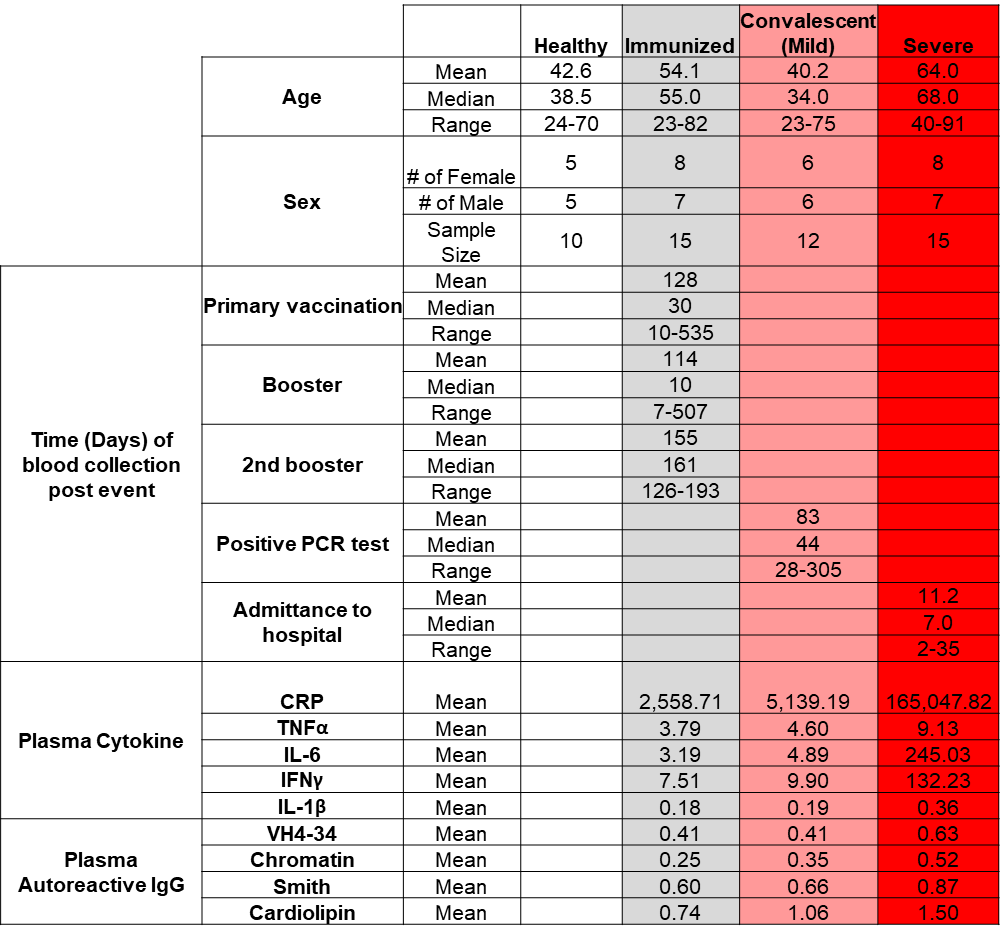


**Supplemental Table 1. Demographics, levels of systemic cytokines and levels of autoreactive antibodies in subjects examined in this study.** Age (mean, median, range) at time of blood draw and sex (# of Females, # of Males, Total Sample size) are detailed in the tables for each patient population. For those immunized against SARS-CoV-2, time (days; mean, median, range) of blood draw post primary vaccination and/or booster are listed. For those with mild SARS-CoV-2 infection, time (days; mean, median, range) of blood draw post positive PCR test is listed. For those with severe SARS-CoV-2 infection, time (days; mean, median, range) of blood draw post admittance to hospital are listed. Levels of systemic cytokines (mean: CRP, TNFα, IL-6, IFNγ and IL-1β) are detailed in the tables for each patient population. CRP values are ng/mL and other cytokine values are pg/mL. Absolute titers of systemic autoreactive IgG antibodies (mean: VH4-34, chromatin, smith, cardiolipin) are detailed in the tables for each patient population and values are O.D. 405nm. Patients were excluded from this study if they had a history of solid organ or bone marrow transplants, chronic lung disease, hemoptysis, increased risk for bleeding, pregnancy or who were immunosuppressed.

|  | Relative to Healthy Control | CD69 | CD86 | CD22 | CD72 | BAFFR | FcRL5 |
| --- | --- | --- | --- | --- | --- | --- | --- |
| DN1 | Immunized | ─ | ─ | **↑** | ─ | ─ | ─ |
|  | Mild SARS-CoV-2 | ─ | ─ | ─ | ─ | ─ | ─ |
|  | Severe SARS-CoV-2 | **↑** | ─ | ─ | **↓** | **↓** | ─ |
| DN2 | Immunized | ─ | ─ | ─ | ─ | ─ | ─ |
|  | Mild SARS-CoV-2 | ─ | ─ | ─ | ─ | ─ | ─ |
|  | Severe SARS-CoV-2 | ─ | ─ | ─ | ─ | ─ | ─ |
| DN3 | Immunized | ─ | ─ | ─ | ─ | ─ | ─ |
|  | Mild SARS-CoV-2 | ─ | **↓** | ─ | **↑** | ─ | ─ |
|  | Severe SARS-CoV-2 | **↑** | **↓** | **↑** | ─ | **↓** | ─ |
|  | **Relative to Immunized Individuals** | **CD69** | **CD86** | **CD22** | **CD72** | **BAFFR** | **FcRL5** |
| DN1 | Healthy control | ─ | ─ | **↓** | ─ | ─ | ─ |
|  | Mild SARS-CoV-2 | ─ | ─ | **↓** | ─ | **↓** | ─ |
|  | Severe SARS-CoV-2 | ↑ | ↑ | ↓ | ↓ | ↓ | ─ |
| DN2 | Healthy control | ─ | ─ | ─ | ─ | ─ | ─ |
|  | Mild SARS-CoV-2 | ─ | ─ | ─ | ─ | ─ | ─ |
|  | Severe SARS-CoV-2 | **↑** | ─ | ─ | **↓** | **↓** | ─ |
| DN3 | Healthy control | ─ | ─ | ─ | ─ | ─ | ─ |
|  | Mild SARS-CoV-2 | ─ | ─ | ─ | ─ | ─ | ─ |
|  | Severe SARS-CoV-2 | **↑** | ─ | ─ | ─ | **↓** | **↑** |

**Supplemental Table 2. Summary of significant differences in surface marker expression for each cell type in each group.** The symbols in the table denote the following; (─) indicates no significant difference, (↑) indicates significant upregulation and (↓) indicates significant downregulation relative to healthy controls (top half of table) or relative to immunized individuals (bottom half of table) from data in Figures 2-4.


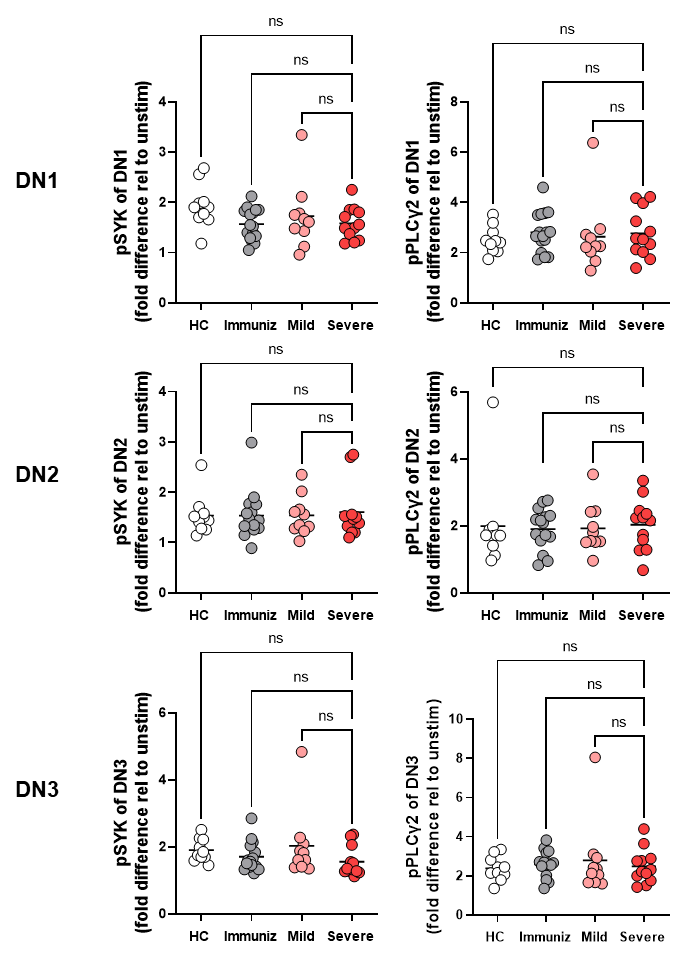


**Supplemental Figure 1. Comparison between cohorts of the degree of BCR signaling in DN subsets.** Fold difference in expression of pSYK or pPLCγ2 between stimulation with 10μg/mL anti-IgG (H+L) F(ab’)2 for 5 min and unstimulated control in DN1, DN2, or DN3 cells from heathy controls (HC, N=10), individuals immunized against SARS-CoV-2 (N=15), or individuals with mild (N=10) or severe SARS-CoV-2 infection (N=12). Statistics. one-way ANOVA, ns not significant.


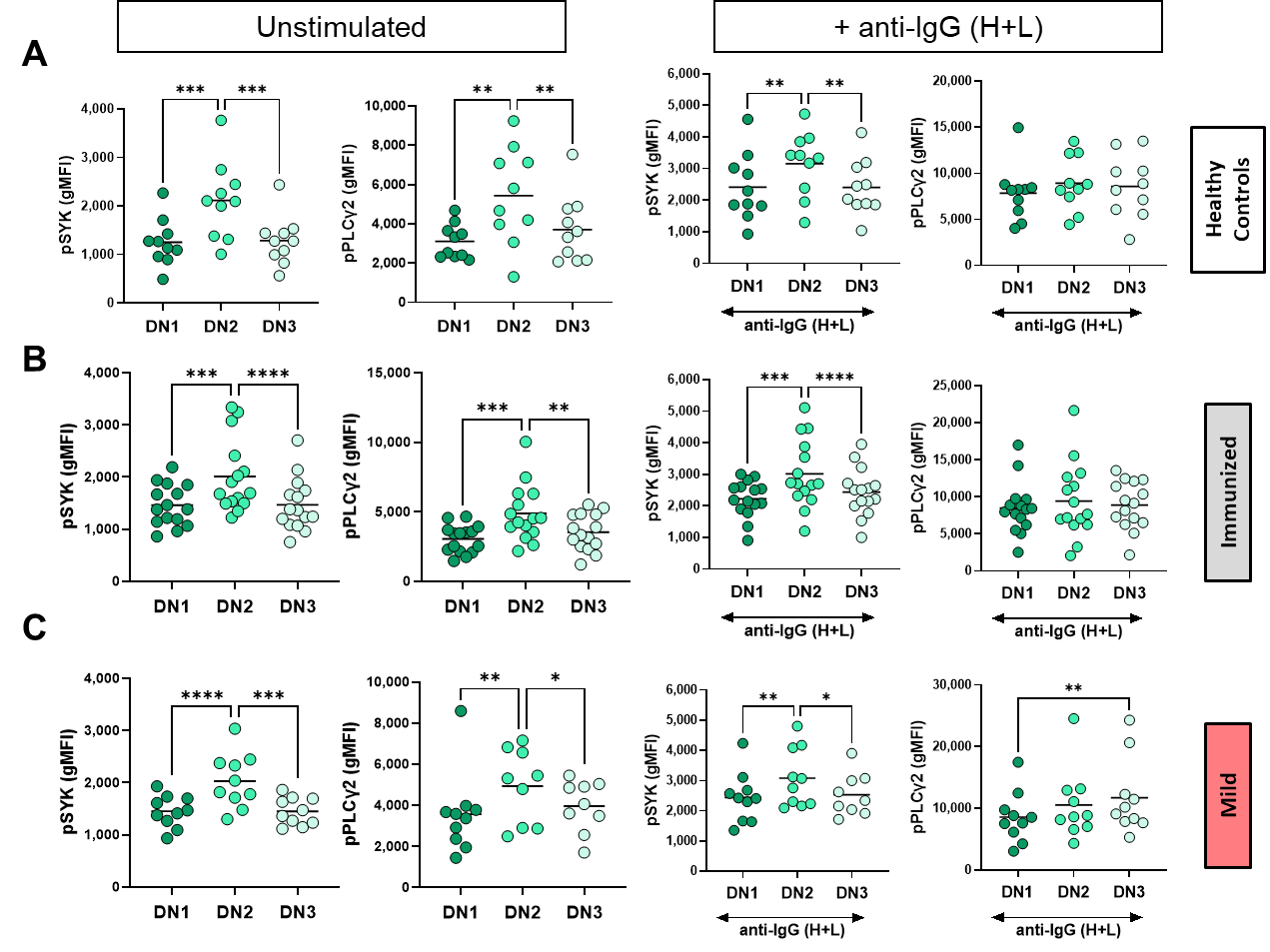


**Supplemental Figure 2.** **Functional comparison of DN1, DN2 and DN3 cells in healthy controls, immunized or mild SARS-CoV-2 infection.** (A) Quantification of expression level of pSYK or pPLCγ2 on DN1, DN2 or DN3 cells from healthy controls (N=10), (B) individuals immunized against SARS-CoV-2 (N=15), or (C) individuals with mild SARS-CoV-2 infection (N=10) at without (unstimulated) or with stimulation by 10μg/mL anti-IgG (H+L) F(ab’)2 for 5 min. Statistics: one-way ANOVA, *p<0.05, **p<0.01, ***p<0.001, ****p<0.0001.


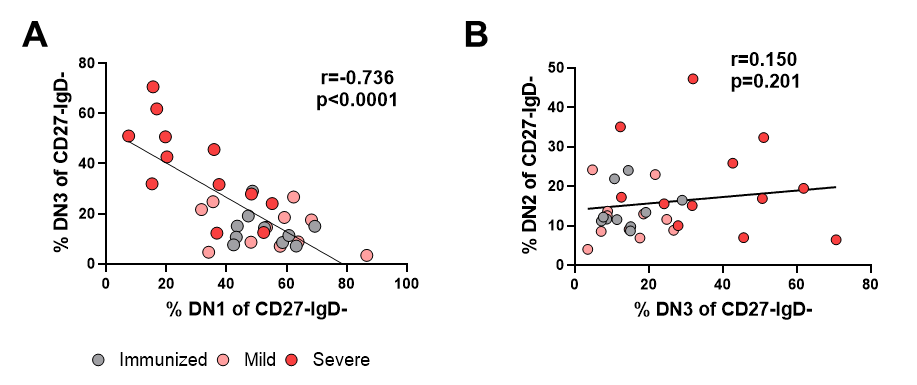


**Supplemental Figure 3.** **Comparison of the frequency of the DN3 subset to the frequency of the DN1 and DN2 subsets.** (A) Correlation of frequency of DN1 cells with the frequency of DN3 cells within total double negative population (CD27-IgD-) or (B) Correlation of frequency of DN3 cells with the frequency of DN2 cells within total double negative population (CD27-IgD-) from individuals immunized against SARS-CoV-2 (N=10), and individuals with mild (N=11) or severe SARS-CoV-2 infection (N=12). Statistics: Pearson correlation, r and p values noted on each panel.
